# The role of *FRIGIDA* and *FLOWERING LOCUS C* genes in flowering time of *Brassica rapa* leafy vegetables

**DOI:** 10.1038/s41598-019-50122-2

**Published:** 2019-09-25

**Authors:** Satoko Takada, Ayasha Akter, Etsuko Itabashi, Namiko Nishida, Daniel J. Shea, Naomi Miyaji, Hasan Mehraj, Kenji Osabe, Motoki Shimizu, Takeshi Takasaki-Yasuda, Tomohiro Kakizaki, Keiichi Okazaki, Elizabeth S. Dennis, Ryo Fujimoto

**Affiliations:** 1 Graduate School of Agricultural Science, Kobe University, Rokkodai, Nada-ku, Kobe, 657-8501 Japan; 2 Institute of Vegetable and Floriculture Science, NARO, Kusawa, Ano, Tsu, Mie 514-2392 Japan; 3 Graduate School of Science and Technology, Niigata University, Ikarashi-ninocho, Niigata, 950-2181 Japan; 4 Plant Epigenetics Unit, Okinawa Institute of Science and Technology Graduate University, Onna-son, Okinawa 904-0495 Japan; 5 Iwate Biotechnology Research Center, Narita, Kitakami, Iwate, 024-0003 Japan; 6 CSIRO Agriculture and Food, Canberra, ACT 2601 Australia; 7 University of Technology, Sydney, PO Box 123, Broadway, NSW 2007 Australia

**Keywords:** Plant molecular biology, Plant breeding

## Abstract

There is a wide variation of flowering time among lines of *Brassica rapa* L. Most *B. rapa* leafy (Chinese cabbage etc.) or root (turnip) vegetables require prolonged cold exposure for flowering, known as vernalization. Premature bolting caused by low temperature leads to a reduction in the yield/quality of these *B. rapa* vegetables. Therefore, high bolting resistance is an important breeding trait, and understanding the molecular mechanism of vernalization is necessary to achieve this goal. In this study, we demonstrated that *BrFRIb* functions as an activator of *BrFLC* in *B. rapa*. We showed a positive correlation between the steady state expression levels of the sum of the *BrFLC* paralogs and the days to flowering after four weeks of cold treatment, suggesting that this is an indicator of the vernalization requirement. We indicate that *BrFLCs* are repressed by the accumulation of H3K27me3 and that the spreading of H3K27me3 promotes stable *FLC* repression. However, there was no clear relationship between the level of H3K27me3 in the *BrFLC* and the vernalization requirement. We also showed that if there was a high vernalization requirement, the rate of repression of *BrFLC1* expression following prolonged cold treatments was lower.

## Introduction

Flowering is an event that transitions a plant from vegetative to reproductive growth, and is regulated by both internal and external factors^1,2^. Because plants use the energy accumulated during the vegetative growth period for the reproductive growth phase to propagate offspring, flowering is a crucial developmental process in a plant’s life cycle^1,2^. Flowering time is also important for the yield of crops or vegetables, and the regulation of flowering time is an important goal of plant breeding^2,3^. Changes to flowering time can broaden the area or the period of suitable cultivation, and lead to tolerance against changing climatic conditions^1,2^.

Many plant species require prolonged cold exposure, generally encountered during the course of winter, before flowering and setting seed. Without exposure to a prolonged cold period, flowering is blocked. This process is known as vernalization, which is derived from the Latin word *vernalis*, meaning ‘of, relating to, or occurring in the spring’^4^. Variation in the requirement for vernalization exists in plant species^1,5,6^. A vernalization requirement is an evolutionary adaptation to temperate climates, preventing flowering before encountering a winter season and ensuring flowering occurs under the more favorable weather conditions of spring^1,2,6^. Vernalization requirement is also important for the quantity and quality of crop production^1,2,6^. In vegetative crops, early bolting and flowering caused by a low vernalization requirement can limit the potential for increase in yield or devalue the products^2,3^.

The molecular mechanism of vernalization has been studied extensively in *Arabidopsis thaliana*, and an abundance of information about its mechanism has been discovered. In *A. thaliana*, the two genes, *FRIGIDA* (*FRI*)^7^ and *FLOWERING LOCUS C* (*FLC*)^8–10^, are the major determinants of flowering time^1,5^. *FRI* is one of the causative genes of natural variation of vernalization in *A. thaliana*, and FRI acts as a positive regulator of *FLC*^7^. Another flowering regulator, *FLC*, encodes a MADS-box transcription factor and acts as a floral repressor^8–10^. *FLC* is expressed before cold exposure and its expression is repressed by vernalization^11^. Cold exposure induces the formation of a plant homeodomain-polycomb repressive complex 2 (PHD-PRC2) that results in an increased abundance of tri-methylation of the 27^th^ lysine of histone H3 (H3K27me3) at the nucleation region of the *FLC* locus^12,13^. Upon return to warm conditions, H3K27me3 spreads over the entire *FLC* gene and silencing of *FLC* is maintained^14,15^.

Varieties of *Brassica rapa* L. include Chinese cabbage (var. *pekinensis*), pak choi (var. *chinensis*), komatsuna (var. *perviridis*), turnip (var. *rapa*), and oilseed (var. *oleifera*). *B. rapa* is closely related to *A. thaliana*, both being members of the *Brassicaceae* family. Bolting caused by low temperature leads to a reduction in the yield and quality of the harvested products of leafy vegetables such as Chinese cabbage, pak choi, and komatsuna or root vegetables such as turnip. Therefore, a line highly resistant to bolting (i.e., possessing a high vernalization requirement) is desirable for the breeding of *B. rapa* cultivars^2,3^. Comparative genetic and physical mapping and genome sequencing studies have revealed that the *B. rapa* genome has undergone a whole-genome triplication, which results in multiple copies of paralogous genes^16–18^. Flowering time genes have been characterized and there are two *FRI* paralogs in *B. rapa*^19,20^. *B. rapa* has four *FLC* paralogs (*BrFLC1*, *BrFLC2*, *BrFLC3*, *BrFLC5*)^19–21^, of which *BrFLC5* is a pseudogene in the reference genome due to the deletion of two exons^16,21^. *BrFLC* genes are expressed before vernalization and their expression is repressed following vernalization^2,3,22^. The silencing of the three functional *BrFLC* paralogs is associated with increased H3K27me3 around the transcription start site^22^. *FLC* paralogs co-localized with quantitative trait loci (QTLs) for flowering time in *B. rapa*^23–27^. Thus, *FLC* genes are considered to be key regulators of the vernalization requirement in *B. rapa*^2,3^.

In *B. rapa*, *FRI* gene function has not yet been confirmed, and how the multiple *FLC* genes are involved in the vernalization requirements is not fully understood. In this study, we characterized two *BrFRI* genes, *BrFRIa* and *BrFRIb*, and confirmed *BrFRIb* functions as an activator of *FLC*. The relationship between expression levels of *BrFRI*s (*BrFRIa* + *BrFRIb*) or *BrFLC*s (*BrFLC1* + *BrFLC2* + *BrFLC3* + *BrFLC5*) and days to flowering was examined. We also examined the expression levels of *BrFLC* genes and the accumulation of H3K27me3, before and after prolonged cold treatment, in two lines that vary in their vernalization requirements. Our results suggest that the steady state of the sum of functional *BrFLC* expression levels and the level of reduction of this expression by vernalization are key factors in determining the vernalization requirement in *B. rapa*.

## Methods

### Plant materials and growth conditions

Nine *B. rapa* lines (RJKB-T02, RJKB-T17, ‘Harunosaiten’, ‘Harusakari’, ‘Natsumaki 50nichi’, ‘Yellow sarson’, BRA2209, Homei, and Osome) were used as plant materials to examine days to flowering after four weeks of cold treatment (Supplementary Table 1). In total, 37 *B. rapa* lines including the above nine lines were used for sequence determination of *BrFRI* genes. Genetic distances among 33 of the 37 lines have been examined^28^ and these 33 lines all need vernalization for flowering (Supplementary Fig. 1, Supplementary Table 1).

Seeds were surface sterilized and grown on agar solidified Murashige and Skoog (MS) plates with 1% (w/v) sucrose under long day (LD) conditions (16 h light) at 22 °C. For vernalizing cold treatments, 14-day seedlings on MS plates were treated for 2, 3, 4, 5, 6, or 8 weeks at 4 °C under LD conditions (16 h light) or four weeks at 4 °C and then seven days in normal growth condition.

To examine the flowering time in the nine lines, seeds were surface sterilized and grown on MS plates with 1% (w/v) sucrose under LD conditions (16 h light) at 22 °C for 14 days, and 14-day seedlings on MS plates were treated for four weeks at 4 °C under LD conditions (16 h light). After cold treatment, the plants were transferred to soil and grown in normal growth conditions. The number of days until the appearance of flower buds was counted and scores were set based on the criteria shown in Supplementary Table 2. More than ten plants of each line were used for examining the flowering time.

### RNA extraction and RT-PCR/qPCR

Total RNA was isolated from 1^st^ and 2^nd^ leaves using the SV Total RNA Isolation System (Promega). The cDNA was synthesized from 500 ng total RNA using ReverTra Ace qPCR RT Master Mix with gDNA Remover (Toyobo). For RT-PCR, the cDNA was amplified using Quick Taq® HS DyeMix (Toyobo). PCR was performed using the following conditions; 1 cycle of 94 °C for 2 min, 25, 30, or 35 cycles of 94 °C for 30 s, 58 °C for 30 s, and 68 °C for 30 s. Primer sequences used for RT-PCR are shown in Supplementary Table 3.

RT-qPCR was performed using LightCycler 96 (Roche). cDNA was amplified using FastStart Essential DNA Green Master (Roche). PCR conditions were 95 °C for 10 min followed by 40 cycles of 95 °C for 10 s, 60 °C for 10 s, and 72 °C for 15 s, and Melting program (60 °C to 95 °C at 0.1 °C/s). After amplification cycles, each reaction was subjected to melt temperature analysis to confirm single amplified products. The expression level of each gene relative to *BrACTIN*^29^ was automatically calculated using automatic CQ calling according to the manufacturer’s instructions (Roche). Data presented are the average and standard error (s.e.) calculated from three biological and experimental replications. Primer sequences used for RT-qPCR are shown in Supplementary Table 3.

### Sequencing DNA fragments of *BrFRI* and *BrFLC* genes

The region covering *BrFRIa* or *BrFRIb* was amplified using primers, FRIa-F1/-R1 or FRIb-F1/-R1, respectively, using genomic DNA as templates. DNAs from 37 *B. rapa* lines were used for the direct sequencing of PCR products (Supplementary Table 1). PCR products were treated by illustra ExoProStar (GE Healthcare Life Sciences) and were sequenced using ABI Prism 3130 (Applied Biosystems). Primer sequences used for direct sequencing are shown in Supplementary Table 3.

Regions covering the coding sequence of each *BrFLC* paralog in RJKB-T02, RJKB-T17, RJKB-T24, Homei, ‘Harunosaiten’, and BRA2209, were amplified using cDNA as templates (Supplementary Table 1). PCR products were treated by illustra ExoProStar (GE Healthcare Life Sciences) and were sequenced using ABI Prism 3130 (Applied Biosystems). PCR was performed using the following conditions; 1 cycle of 94 °C for 2 min, 35 cycles of 94 °C for 30 s, 58 °C for 30 s, and 68 °C for 30 s. Primer sequences used for direct sequencing are shown in Supplementary Table 3.

The genomic regions covering *BrFLC1*, *BrFLC2*, and *BrFLC3* and their promoter regions were amplified using genomic DNA as a template in BRA2209. PCR products were then cloned into pGEM®-T Easy vector (Promega). Nucleotide sequences of three clones of PCR products were determined with the ABI Prism 3130 (Applied Biosystems), and the data were analyzed using Sequencher (Gene Codes Corporation, MI, USA). PCR was performed using the following conditions; 1 cycle of 94 °C for 2 min, 35 cycles of 94 °C for 30 s, 58 °C for 30 s, and 68 °C for 2 min. Primer sequences and their positions used for PCR and sequencing are shown in Supplementary Fig. 2 and Supplementary Table 3.

### Constructs and plant transformation

Using cDNAs from leaves of RJKB-T24, either *BrFRIb* or *BrFLC1, 2*, or 3 cDNA fragments were amplified by RT-PCR using primers designed to add *Bam* HI and *Sac* I restriction sites to the 5′- and 3′-ends (Supplementary Table 3), and PCR products were then cloned into pGEM®-T Easy vector (Promega). DNA fragments of *BrFRIb* or *BrFLC1*, *2*, or 3 cDNA was inserted into *Bam* HI and *Sac* I restriction sites of pBI121. These constructs were transformed into *Agrobacterium tumefaciens* strain EHA105, and transformation of Columbia-0 (Col) accession in *A. thaliana* was carried out by the floral dip procedure^30^. Transgenic seedlings were selected through resistance to kanamycin on a selection medium.

Seeds of T_2_ plants were sown on MS medium with or without four weeks of cold treatment and grown under LD conditions (16 h light) at 22 °C. After growing plants on MS medium, they were transferred to soil and grown under the conditions described above. Flowering time in *A. thaliana* was expressed as the number of rosette leaves at the time of flowering.

### Chromatin immunoprecipitation (ChIP)

ChIP experiments were performed as described by Buzas *et al*.^31^. One gram of non-crosslinked chromatin taken from the 1^st^ and 2^nd^ leaves of Homei and ‘Harunosaiten’ was used (Supplementary Table 1). Mononucleosomes were obtained by MNase digestion and samples were sonicated twice. The samples were incubated with anti-H3K27me3 (Millipore, 07-449) antibodies for 4 h and then with protein A agarose for 2 h at 4 °C with rotation. The protein A agarose was washed, and immunoprecipitated DNAs were eluted by proteinase K treatment followed by a clean-up using Qiagen PCR cleanup kit (Qiagen). We validated the enrichment of purified immunoprecipitated DNAs by ChIP-qPCR using the previously developed positive and negative control primer sets of H3K27me3 (Supplementary Table 3)^22^. Three independent ChIP experiments were carried out on each sample for biological replicates.

ChIP-qPCR was performed by the same method as the RT-qPCR using the immunoprecipitated DNA as a template. The H3K27me3 level of each *BrFLC* gene relative to the *SHOOT MERISTEMLESS* gene (*BrSTM*)^22^, which has H3K27me3 accumulation, was automatically calculated using automatic CQ calling according to the manufacturer’s instructions (Roche). The difference in the amplification efficiency between primer pairs was corrected by calculating the difference observed by qPCR amplifying the input-DNA as a template. Data presented are the average and standard error (s.e.) from three biological and experimental replications. Primer sequences used for ChIP-qPCR are shown in Supplementary Table 3.

### Amino acid sequence analysis

Using the genome sequences of *BrFRIa* and *BrFRIb* in 37 *B. rapa* lines, predicted amino acid sequences were obtained. The amino acid sequences of BrFRIa and BrFRIb in 37 lines of *B. rapa*, two BoFRI^32^, and AtFRI (AF228499.1) were aligned using ClustalW (http://www.ddbj.nig.ac.jp/search/clustalw-j.html). A phylogenetic tree was constructed with the neighbor joining method^33^, and the bootstrap probabilities of 1,000 trials were calculated.

## Results

### Variation of the days to flower after prolonged cold treatment

To determine the duration of prolonged cold treatment, we determined the days to flowering after prolonged cold treatment. We examined the percentage of plants that had flowered in two early (Homei and RJKB-T02) and two late flowering lines (RJKB-T17 and BRA2209) with different durations of cold treatments, i.e., two, three, four, or five weeks. After two weeks of cold treatment, no line flowered within 100 days (Supplementary Fig. 3). All plants of two early flowering lines flowered after three weeks of cold treatment, while no plant of two late flowering lines flowered (Supplementary Fig. 3). In two late flowering lines, four of six plants of RJKB-T17 flowered following four weeks of cold treatment, while no plant in BRA2209 flowered following four weeks of cold treatment (Supplementary Fig. 3). Following five weeks of cold treatment, all lines flowered even though there were differences in days to flowering (Supplementary Fig. 3). From these data, we determined that four weeks of cold treatment is suitable for detecting differences in vernalization requirement among the *B. rapa* lines.

**Figure 1:**
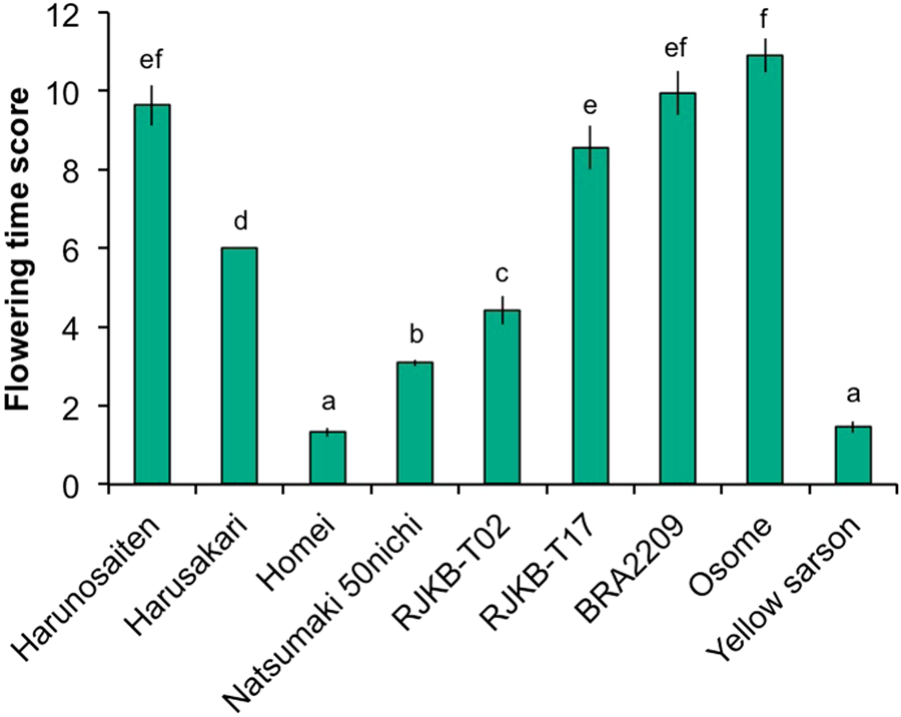
Flowering time score represented by the expected value of number of days from sowing to bolting in nine *B. rapa* lines. Data presented are the average and standard error (s.e.) from more than ten plants. Letters above the bars indicate significant differences at *p* < 0.05 (Tukey-Kramer test).

Next, we examined the days to flower after four weeks of cold treatment in nine *B. rapa* lines. Scores were used for the evaluation of flowering time, because some plants in the late flowering line did not flower within 100 days (see Methods). ‘Yellow Sarson’ and Homei were early flowering, while Osome, BRA2209, RJKB-T17, and ‘Harunosaiten’ were late flowering (Fig. 1).

### Functional analysis of *FRIGIDA* in *B. rapa*

As the early flowering phenotype in some *A. thaliana* accessions is due to the loss of function of *AtFRI*, we examined the sequence variation in *BrFRI* genes using 37 lines of *B. rapa* including the nine lines whose flowering time had been assessed (Fig. 1, Supplementary Table 1). In the reference genome, Chiifu-401-42, there are two *FRI* genes, *BrFRIa* (Bra029192, A03) and *BrFRIb* (Bra035723, A10). The *BrFRIa* sequence in the reference genome is comprised of three exons and showed sequence similarity to *AtFRI* and *BoFRIa*. BoFRIa^32^ and AtFRI^7^ have already been shown to be functional activators of *FLC*, considering that *BrFRIa* in Chiifu-401-42 is functional; sharing a high sequence similarity to BoFRIa suggests that BrFRIa may perform a similar function. The nucleotide sequence of *BrFRIa* in 37 lines was determined by direct sequencing. There was a high sequence similarity of the amino acid sequence of BrFRIa among 37 *B. rapa* lines (from 97.8% to 100.0%) (Supplementary Table 4). The amino acid sequence identities between BrFRIa and AtFRI were from 56.8% to 57.5% and from 87.9% to 88.1% between BrFRIa and BoFRIa (Supplementary Figs 4, 5, Supplementary Table 4). Lines showing different flowering times had identical amino acid sequences of BrFRIa (Fig. 1, Supplementary Table 4), indicating that the differences of vernalization requirements among the nine *B. rapa* lines are not due to amino acid sequence variation in BrFRIa.

In contrast, the annotated *BrFRIb* (Bra035723), termed *BrFRIbΔ*, is comprised of two exons, and appears to lack the 3^rd^ exon (Supplementary Fig. 6). We mapped RNA-seq reads that was previously performed^34^ using 14-day leaves in RJKB-T23 and RJKB-T24 on the region covering *BrFRIbΔ* and found another ORF, which contains three exons (Supplementary Fig. 7), suggesting that an unannotated functional copy of *BrFRIb* is present in the reference genome sequence. As further evidence of this, transformation of the annotated *BrFRIbΔ* driven with the 35S CaMV promoter into the Col accession of *A. thaliana*, which lacks AtFRI function, did not complement the AtFRI function (Supplementary Fig. 8), indicating that the annotated BrFRIbΔ is non-functional.

**Figure 2:**
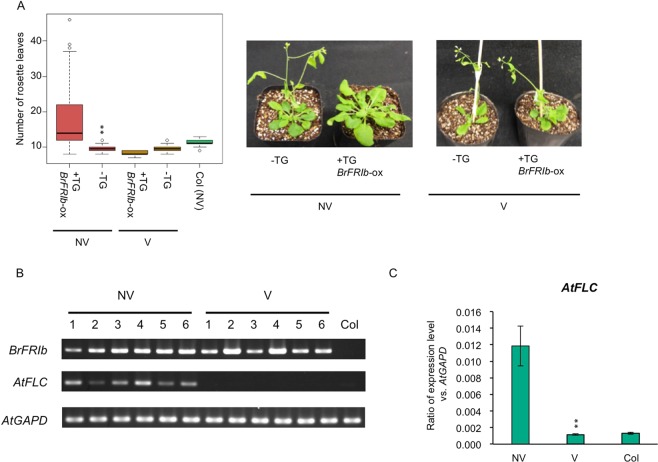
Overexpression of *BrFRIb* causes late flowering and induce *AtFLC* expression. (**A**) Number of rosette leaves and flowering-time phenotypes in T_2_ plants with overexpressing *BrFRIb* with (V) or without vernalization (NV). +TG and −TG show the presence and absence of transgenes (TG), respectively. **p < 0.01 (Students *t*-test) (**B**) RT-PCR analysis showing transcription of *BrFRIb* and *AtFLC* before and after four weeks of cold treatment. Non-vernalized Col line is included as a control. *AtGAPD* was used as a control to demonstrate equal RNA loading. NV, non-vernalized; V, vernalized. (**C**) RT-qPCR analysis of *AtFLC* with (V) and without (NV) four weeks of cold treatment. *AtFLC* expression level relative to *AtGAPD* is shown in the y-axis. Non-vernalized Col line is included as a control. Data presented are the average and standard error (s.e.) from three biological and experimental replications. **p < 0.01 (Students *t*-test).

We examined whether the newly identified BrFRIb in this study is functional. We transformed *BrFRIb* into the Col accession of *A. thaliana*, and 14 independent T_1_ plants were obtained. The flowering time segregated in T_2_ plants that were derived from three independent T_1_ plants, and the flowering times of T_2_ plants with the transgene were later than the T_2_ plants without the transgene or wild type Col (Student *t*-test, *p* < 0.01) (Fig. 2A). We also found that T_2_ plants from some T_1_ lines did not flower even when the rosette leaf number was greater than 45. We confirmed the induction of *AtFLC* expression in these late flowering transgenic plants (Fig. 2B). T_2_ seeds were treated with cold for four weeks, and then examined for flowering time. The flowering time in T_2_ plants with the transgene was the same as without the transgene (Fig. 2A). Repression of *AtFLC* by cold treatment was observed in T_2_ plants with the transgene (Fig. 2B,C), indicating that *AtFLC* induced by *BrFRIb* is suppressed by cold treatment. These results indicate that BrFRIb functioned like AtFRI.

We determined the nucleotide sequence of *BrFRIb* in 37 lines by direct sequencing. There was a high sequence similarity of the amino acid sequence of BrFRIb among *B. rapa* lines (from 95.8% to 100.0%) (Supplementary Fig. 5, Supplementary Table 4). The amino acid sequence identity between BrFRIb and AtFRI were from 59.4% to 59.9% and from 85.8% to 87.4% between BrFRIb and BoFRIb (Supplementary Figs 4, 5, Supplementary Table 4). The amino acid sequence identities ranged from 63.1% to 64.3% between BrFRIa and BrFRIb (Supplementary Table 4). Like BrFRIa, lines showing different flowering time had identical amino acid sequences of BrFRIb (Fig. 1, Supplementary Table 4), indicating that the difference of vernalization requirement among nine *B. rapa* lines is not due to the amino acid sequence variation of BrFRIb.

**Figure 3:**
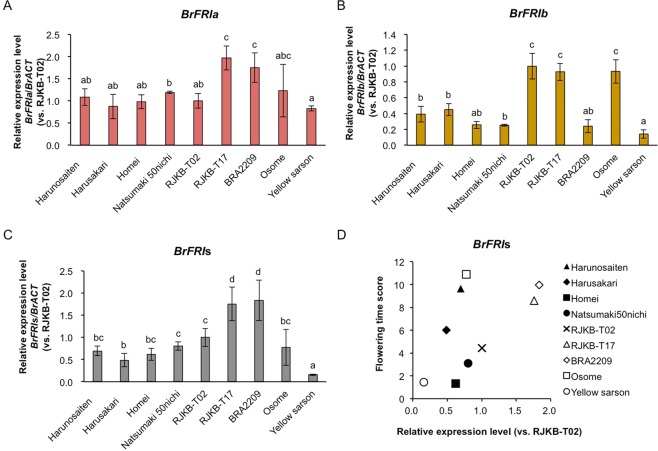
The relationship between the expression levels of *BrFRI* and flowering time. (**A**–**C**) There is variation of the expression levels of *BrFRIa, BrFRIb*, or *BrFRI*s (*BrFRIa* + *BrFRIb*) among nine *B. rapa* lines. Expression level of each gene relative to *BrACTIN* (*BrACT*) is calculated, and the y-axis shows the ratio against RJKB-T02. Data presented are the average and standard error (s.e.) from three biological and experimental replications. Letters above the bars indicate significant differences at *p* < 0.05 (Tukey-Kramer test). (**D**) The relationship between the expression levels of *BrFRI*s and flowering time score in nine *B. rapa* lines.

Next, we examined whether transcription levels of *BrFRI* genes contribute to the difference of vernalization requirement. We examined the transcription levels of *BrFRIa, BrFRIb*, or *BrFRI*s (*BrFRIa* + *BrFRIb*) by RT-qPCR in 14-day leaves of the nine lines whose flowering time had been measured (Figs 1 and 3A–C). Expression in ‘Yellow sarson’ was the lowest, while RJKB-T17 and BRA2209 had the highest expression levels of *BrFRI*s, with expression levels in BRA2209 being 6.8 times higher than that in ‘Yellow sarson’ (Fig. 3C). There was a moderate correlation between *BrFRI*s expression level and flowering time but it was not statistically significant (*r* = 0.56, *p* > 0.05) (Fig. 3D). There was no correlation between *BrFRIa* or *BrFRIb* expression level and flowering time (Supplementary Fig. 9).

### Three FLOWERING LOCUS C paralogs function as floral repressors in *B. rapa*

We examined whether *BrFLC* is a key regulator of the differences in vernalization requirements for *B. rapa*. First, we confirmed all three BrFLCs (*BrFLC1*, *BrFLC2*, and *BrFLC3*) function as floral repressors. Transformation of a 35 S promoter::*BrFLC1*cDNA, 35 S promoter::*BrFLC2*cDNA, or 35 S promoter::*BrFLC3*cDNA construct into the Col accession of *A. thaliana*, where *AtFLC* was not expressed because of loss of function of AtFRI, revealed that transgenic plants with overexpressed *BrFLC1*, *BrFLC2*, or *BrFLC3* showed late flowering (Supplementary Fig. 10), confirming that all three BrFLCs function as floral repressors like AtFLC.

Second, we examined the amino acid sequences of three functional BrFLC paralogs (BrFLC1, BrFLC2, and BrFLC3) in RJKB-T24, which was the line used for testing the 35 S promoter::*BrFLC* constructs. The amino acid sequence of the early flowering lines, RJKB-T02 and Homei, and the late flowering lines, RJKB-T17, ‘Harunosaiten’, and BRA2209, were also examined. A comparison of the amino acid sequences for each BrFLC paralog showed no sequence differences between lines, indicating that any difference in flowering time is not due to amino acid sequence variation.

**Figure 4:**
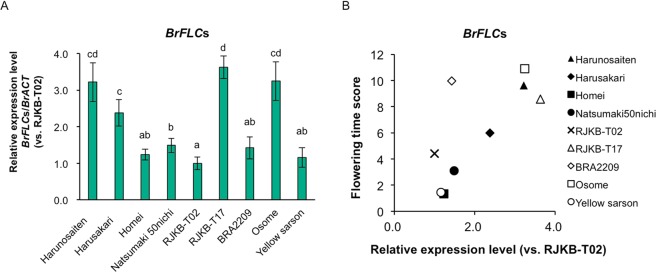
The relationship between the expression level of *BrFLCs* and flowering time. (**A**) There is variation of the expression levels of *BrFLC*s (*BrFLC1* + *BrFLC2* + *BrFLC3* + *BrFLC5*) among nine *B. rapa* lines. Expression level of each gene relative to *BrACTIN* (*BrACT*) is calculated, and the y-axis shows the ratio against RJKB-T02. Data presented are the average and standard error (s.e.) from three biological and experimental replications. Letters above the bars indicate significant differences at *p* < 0.05 (Tukey-Kramer test). (**B**) The steady state expression level of *BrFLC*s is associated with days to flower after four weeks of cold treatment. The correlation coefficient between *BrFLC*s and flowering time score is 0.73 (*p* < 0.05) and if remove the BRA2209 data (outlier) being 0.91 (*p* < 0.05).

Third, we examined the expression levels of *BrFLC* genes in the nine *B. rapa* lines whose flowering time had been measured (Fig. 1, Supplementary Table 1) using a primer set that can amplify all four *FLC* genes. The lowest level of *BrFLC*s was in early flowering RJKB-T02 and the highest in late flowering RJKB-T17; the expression level in RJKB-T17 was 3.6 times higher than in RJKB-T02 (Fig. 4A). The expression levels of *BrFRI*s and *BrFLC*s showed a weak correlation (*r* = 0.23, *p* > 0.05) (Supplementary Fig. 11). There was a high correlation between *BrFLC*s expression level and flowering time (*r* = 0.73, *p* < 0.05) (Fig. 4B), suggesting that the expression level of *BrFLC*s before cold treatment is associated with the vernalization requirement.

### Variation of the vernalization response in *BrFLC*s

**Figure 5:**
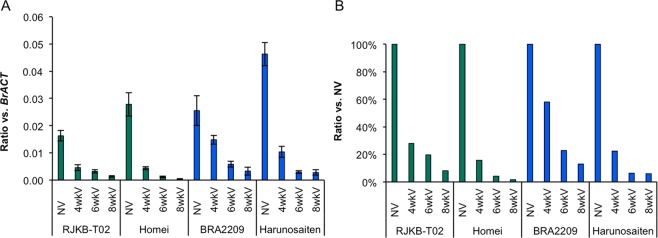
Variation of *BrFLC* repression by cold treatment. (**A**) Expression pattern of *BrFLC*s (*BrFLC1* + *BrFLC2* + *BrFLC3* + *BrFLC5*) in four *B. rapa* lines before (NV) and after 4, 6, and 8 weeks of cold treatments (4wkV, 6wkV, and 8wkV, respectively). Y-axis represents the relative expression level of *BrFLC*s compared to *BrACTIN* (*BrACT*). Data presented are the average and standard error (s.e.) from three biological and experimental replications. (**B**) The ratio of the expression level after cold treatment compared to before cold treatment.

Of nine *B. rapa* lines whose flowering time had been measured, two early (RJKB-T02, Homei) and two late flowering (BRA2209, ‘Harunosaiten’) lines were selected to examine the *BrFLC*s expression in different durations of cold treatments. A decrease in *BrFLC*s expression levels in response to four weeks of cold treatment was from 15.8% to 47.8%, with the weakest repression observed in BRA2209 (Fig. 5A,B). The rate of repression of *BrFLC*s expression by four weeks of cold treatment was not related to the expression level of *BrFLC*s before cold treatment (Fig. 5A,B). *BrFLC* expression levels after four weeks of cold treatment in BRA2209 and ‘Harunosaiten’ were higher than that in RJKB-T02 and Homei (*p* < 0.05; Tukey-Kramer test) (Fig. 5A). This difference was related to the difference of flowering time after four weeks of cold treatment (Fig. 1). *BrFLC*s expression levels were reduced following the cold treatment length in all four lines (Fig. 5A). The rate of decrease in *BrFLC*s expression level was lowest in BRA2209 (Fig. 5B).

### Histone modification spreads at the *BrFLC* locus upon a return to normal growth conditions after vernalization

We selected two lines (Homei, ‘Harunosaiten’) to examine the relationship between H3K27me3 levels at the *BrFLC* loci and differences in the vernalization requirements. Homei showed low levels of *BrFLC*s expression before cold treatment and an early flowering phenotype after four weeks of cold treatment, whereas ‘Harunosaiten’ showed high levels of *BrFLC*s expression before cold treatment and late flowering phenotype after four weeks of cold treatment (Figs 1 and 5). In both lines, the expression levels of *BrFLC*s decreased following the four weeks of cold treatment and transcriptional repression was maintained upon return to normal temperature (Supplementary Fig. 12).

**Figure 6:**
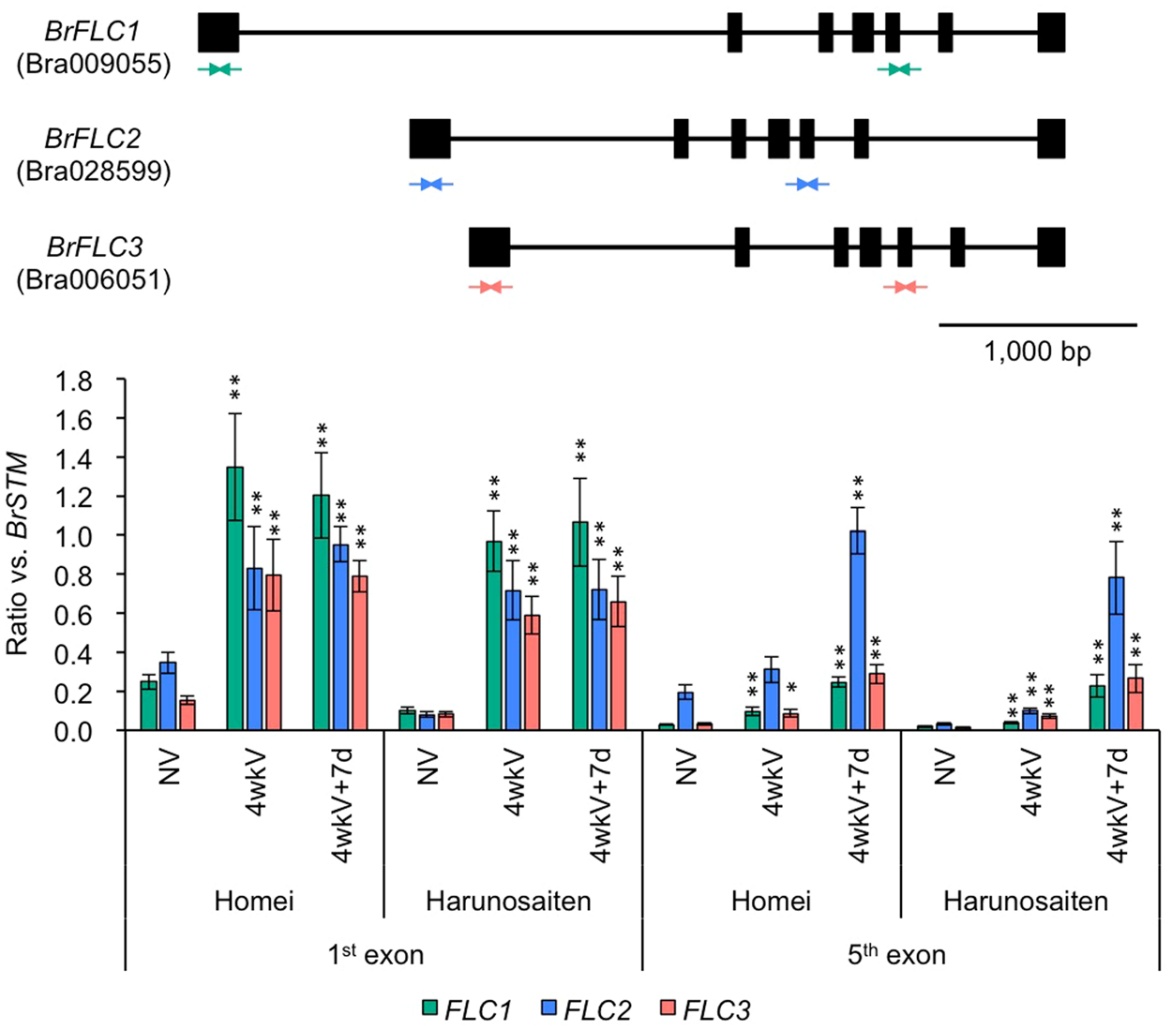
ChIP-qPCR using H3K27me3 antibodies of *BrFLC* genes before and after four weeks of cold treatment. Upper panel is the gene structure of three *BrFLC* paralogs. Black boxes represent exon and arrows represent the primer position for ChIP-qPCR. Bottom panel shows the level of H3K27me3 in three *BrFLC*s before and after four weeks of cold treatment. Y-axis represents the ratio compared to *BrSTM*, which is an H3K27me3-marked gene. Data presented are the average and standard error (s.e.) from three biological and experimental replications. Statistical tests between NV and 4wkV or between NV and 4wkV + 7d are shown (Student *t*-test, *p < 0.05, **p < 0.01). NV, non-vernalized; 4wkV, four weeks of cold treatment; 4wkV + 7d, four weeks of cold treatment and then seven days normal growth condition.

At the end of four weeks of cold treatment, H3K27me3 accumulation was observed around the transcription start site (TSS) of *BrFLC* in both lines. The accumulation of H3K27me3 levels in the region around the TSS was maintained in both lines seven days after returning to normal growth conditions (Fig. 6). In the 5^th^ exon regions, H3K27me3 levels slightly increased, but were lower relative to the TSS in both lines at the end of four weeks of cold treatment (Fig. 6). In both lines, H3K27me3 levels increased seven days after returning to normal growth conditions (Fig. 6); the spreading of H3K27me3 regions in the *BrFLC* loci was observed in Homei and ‘Harunosaiten’ after seven days of normal growth conditions following four weeks of cold treatment (Fig. 6). The accumulation of H3K27me3 was similar between Homei and ‘Harunosaiten’, suggesting that the level of H3K27me3 at the *BrFLC* loci does not explain the difference in vernalization requirement between these lines.

### Characterization of three functional *BrFLC* paralogs in BRA2209

**Figure 7:**
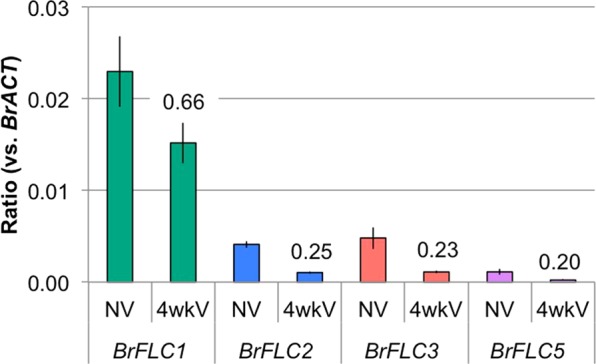
Expression pattern of *BrFLC* genes in BRA2209 before (NV) and after four weeks of cold treatments (4wkV). Expression level of each *BrFLC* paralog relative to *BrACTIN* (*BrACT*) is calculated. Data presented are the average and standard error (s.e.) from three biological and experimental replications. The ratio of the expression level after cold treatment compared to before cold treatment are shown above the bars. NV, non-vernalized; 4wkV, four weeks of cold treatment.

We found that the rate of repression of *BrFLC*s expression by cold treatment was low in BRA2209, and this line showed a high vernalization requirement (Figs 1 and 5). We examined the expression level in each *BrFLC* paralog in BRA2209 using paralog specific primer sets^26^. Before cold treatment, *BrFLC1* had the highest expression among the four paralogs (Fig. 7). After four weeks of cold treatment, *BrFLC1* still had the highest expression level and the suppression rate of *BrFLC1* following four weeks of cold treatment was lower than that of other *BrFLC* paralogs (Fig. 7).

The sequences of full lengths of the genic regions of *BrFLC1*, *BrFLC2*, and *BrFLC3* in BRA2209 were determined. In *BrFLC1*, there was a 410 bp deletion, including part of the 7^th^ exon (31 bp of the 3’ region including stop codon) and a downstream region (Supplementary Fig. 13). Except for two SNPs in the 5^th^ intron, the other exon and intron regions were identical to the reference sequence. There were three SNPs in the 963 bp region upstream of the TSS, and no sequence differences in the 497 bp region downstream from the deleted region in *BrFLC1* of BRA2209 (Supplementary Fig. 13). In *BrFLC2*, there were several substitutions and indels in promoter and intron regions, but no substitutions in the exon regions (Supplementary Fig. 13). In *BrFLC3*, the promoter region had some sequence differences in comparison to the reference genome and there were some substitutions and indels in the intron regions. However, the coding sequence was identical to the reference genome (Supplementary Fig. 13).

## Discussion

High bolting resistance is an important trait for leafy vegetables in *B. rapa*, and previous reports showed that *FLC* is a key gene for vernalization^2,3^. Co-localization of flowering time QTLs with the *BrFLC1* or *BrFLC2* gene suggests that the loss-of-function of *BrFLC* causes early flowering^2,3^. Our study and a previous study revealed that all three *BrFLC*s function as floral repressors^35^. The loss-of-function of one of the *BrFLC* paralogs can result in early flowering, implying that the expression of *BrFLC* paralogs works to repress flowering in a quantitative manner^2,3^.

From the reference genome sequence of *B. rapa*, two *BrFRI* genes were identified. *BrFRIa* has three exons and is similar to the functional *FRI* genes found in other species, while the annotated *BrFRIb* in the reference genome (*BrFRIbΔ*) has two exons and appears to be truncated in the C-terminus. As the C-terminus is critical in AtFRI function^36^, BrFRIbΔ could be non-functional. Indeed, transformation of *BrFRIbΔ* into the *A. thaliana* Col accession did not complement the early flowering phenotype. However, we found a third exon by mapping RNA-seq reads against the reference genome. Complementation using this new ORF, termed *BrFRIb*, confirmed it to be functional, and transformation of *BrFRIb* into Col delayed flowering. In addition, *BrFRIb* induced *AtFLC* transcription and induced *AtFLC*, which was suppressed by four weeks of cold treatment, indicating that BrFRIb has the same function as AtFRI. We did not find mutations leading to a major defect in the translated protein in any of 37 varieties of *B. rapa*, and the amino acid sequences of BrFRIa or BrFRIb among these varieties were more than 95% identical. In *B. oleracea*, BoFRIa has been confirmed to be functional by a complementation experiment^32^, and has about 88% amino acid sequence identity to BrFRIa, suggesting that BrFRIa is functional. We consider that both BrFRIa and BrFRIb are functional activators of the floral repressor gene *FLC* in *B. rapa*.

In our study, the nine lines of *B. rapa* did not show any positive correlation between the expression levels of the *BrFRI*s and *BrFLC*s before cold treatment. These results suggest there is no strong correlation between the expression levels of *FRI* and *FLC* before vernalization in the genus *Brassica*.

There was variation in the flowering time after four weeks of cold treatment among nine lines of *B. rapa*, suggesting that a cold treatment of four weeks in duration is not saturating for promoting flowering in some lines. In *A. thaliana*, the variation of flowering time is due to naturally occurring loss-of-function mutations, which have originated independently and result in early flowering accessions (summer annual habit)^7,37–40^. It is unlikely that sequence variation in the coding sequences of *BrFRIa* or *BrFRIb* influences flowering time variation or the vernalization requirement, because the amino acid sequences are highly conserved and there were no differences in the amino acid sequence between lines showing different flowering times. The absence of an association between *BrFRI* expression levels and vernalization requirement in this study and the low number of reports showing an association between flowering time QTL and *FRI* in the genus *Brassica*^41^ suggest that the variation of vernalization requirement in *B. rapa* is not greatly influenced by sequence or transcriptional variation of *BrFRI*.

All three *BrFLC*s function as floral repressors; this has been confirmed by other groups in *B. rapa*^35^ or *B. napus*^42^. These results suggest that we should consider not only each paralogous *BrFLC* transcript, but also the sum of the three paralogous *BrFLC* transcripts as an important factor for the vernalization requirement. There is a positive correlation between the expression levels of *BrFLC* paralogs before cold treatment and the days to flowering after four weeks of cold treatment. This suggests that the expression levels of *BrFLC* genes before cold treatment may be an indicator of the duration of cold required for vernalization. The rate of suppression of *BrFLC*s expression by cold treatment was similar among lines except for BRA2209. Generally, if the rate of repression of *BrFLC* expression is similar among varieties, the expression level before cold treatment is predictive of duration of cold required for vernalization. As a longer cold period will be required to suppress *BrFLC*s expression in lines having a higher *BrFLC*s expression prior to cold treatment, the positive correlation between the expression levels of *BrFLC*s before cold treatment and days to flowering after four weeks of cold treatment supports this idea. However, our experiment assessed nine lines, and we need to verify this possibility by analyzing additional lines.

In BRA2209, expression levels of *BrFLC*s before cold treatment were not as high as in other lines, but the rate of repression of *BrFLC*s expression by cold treatment was low, especially of *BrFLC1*, leading to higher expression levels of *BrFLC*s after four weeks of cold treatment, consistent with the late flowering phenotype. An extremely late bolting line of *B. rapa* has a long insertion in the 1^st^ intron of *BrFLC2* and *BrFLC3*, and the rate of decrease in the expression of *BrFLC2* and *BrFLC3* is low, indicating a weak vernalization response^27^. We did not identify any sequence difference in the 1^st^ intron of *BrFLC1* between BRA2209 and the reference genome. In contrast, we found a 401 bp deletion covering part of the 7^th^ exon and downstream regions in *BrFLC1* of BRA2209, suggesting that the 3’ region of *BrFLC1* might include a sequence important for the response to prolonged cold.

We have shown that *FLC* chromatin is enriched with the active histone marks, H3K4me3 and H3K36me3, prior to cold treatment, and that these histone marks are replaced with the repressive histone mark, H3K27me3, during cold exposure^22^, suggesting that chromatin change is important for the repression of *FLC* in the vernalization of *B. rapa*. In *A. thaliana*, increasing the duration of cold quantitatively enhances the stability of *AtFLC* repression, and the necessary period of cold treatment varied among accessions. In two different accessions of *A. thaliana* (*FRI* Col and Lov-1), the accumulation of H3K27me3 at the entire *FLC* locus, upon transfer of the plants back to warm conditions after cold treatment, was faster in the accession that requires a shorter period of cold (*FRI* Col) than in the accession that needs a longer period of cold (Lov-1)^43^. When treated with four weeks of cold, an enrichment of H3K27me3 was observed around the TSS of the *BrFLC* loci, but not at the region covering the 5^th^ exon in either line. Upon returning to warm conditions after cold exposure, H3K27me3 accumulation occurred at both TSS and the 5^th^ exon regions in both lines, suggesting that H3K27me3 spreads from the 5’ to 3’ direction in *BrFLC* genes to maintain *FLC* repression. The spreading of H3K27me3 in the *BrFLC* locus is similar to the spreading reported in *A. thaliana*^14,15,43^. Unlike the distinct difference in H3K27me3 accumulation reported in *A. thaliana*, we did not find a difference in the accumulation patterns of H3K27me3 at the *BrFLC* loci between early and late flowering lines of *B. rapa*.

Taken together, two factors, the steady state expression levels of *BrFLC*s and the sensitivity of the repression of *BrFLC*s by cold treatment, are important for the vernalization requirement in *B. rapa*. Further study will be required to identify whether variations of these two factors are regulated by *cis* or *trans*.

## Supplementary information

Supplementary figures and tables
