# Supplementary material for: The role of FRIGIDA and FLOWERING LOCUS C genes in flowering time of Brassica rapa leafy vegetables
Source: Sci Rep. 2019 Sep 25;9:13843. doi: 10.1038/s41598-019-50122-2 (PMC6761103; doi:10.1038/s41598-019-50122-2)
Supplement: Supplementary file 1 — Supplementary figures and tables [file 41598_2019_50122_MOESM1_ESM.pdf]

Supplementary Information

Title of the manuscript:

The role of *FRIGIDA* and *FLOWERING LOCUS C* genes in flowering time of *Brassica rapa* leafy vegetables

Author list:

Satoko Takada, Ayasha Akter, Etsuko Itabashi, Namiko Nishida, Daniel J. Shea, Naomi Miyaji, Hasan Mehraj, Kenji Osabe, Motoki Shimizu, Takeshi Takasaki-Yasuda, Tomohiro Kakizaki, Keiichi Okazaki, Elizabeth S. Dennis, Ryo Fujimoto

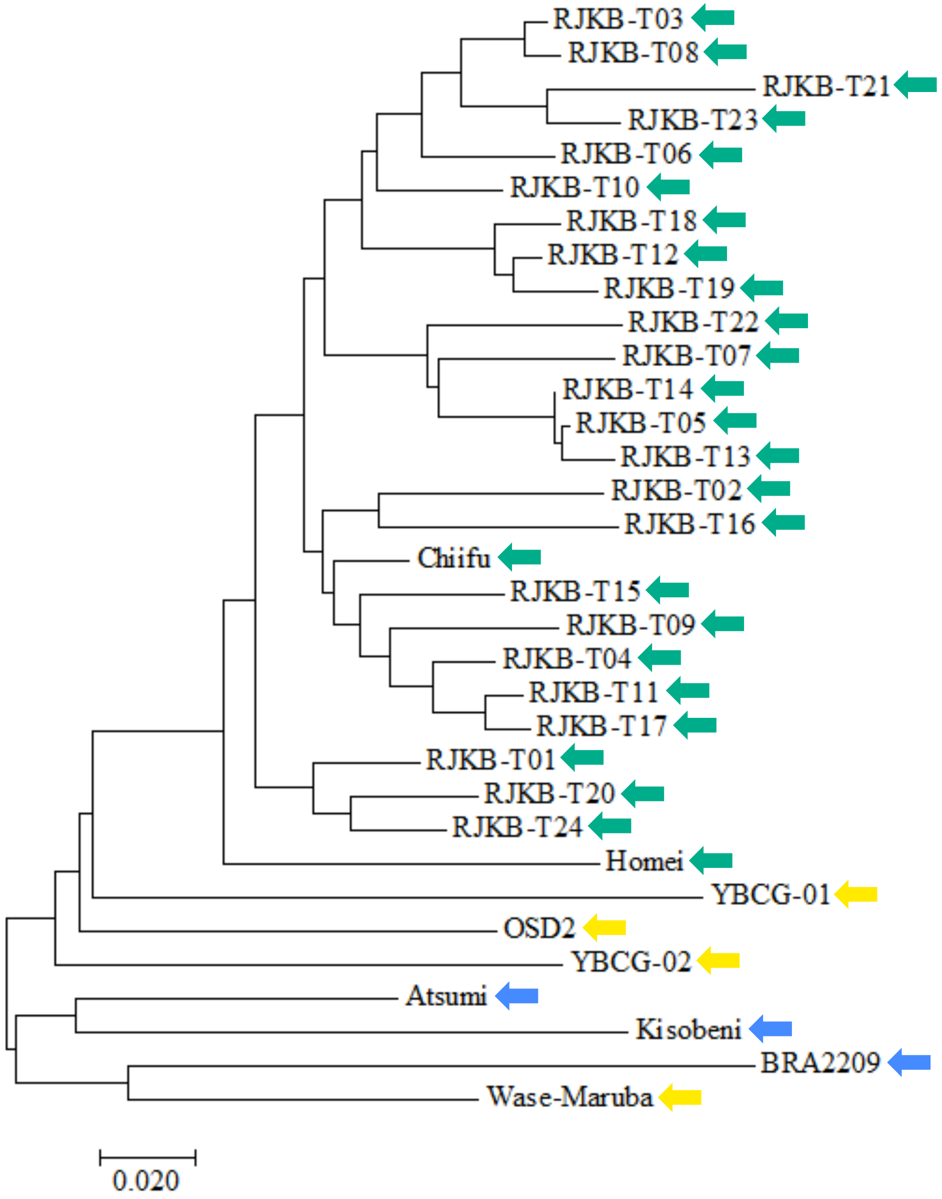

Supplementary Figure 1. A phylogenetic tree of the 33 lines in *B. rapa* based on genetic distance calculated by Kawamura et al. 2016[28]. Additional lines have been included in the analysis. Green, yellow, and blue arrows indicate the Chinese cabbage (var. *pekinensis*), komatsuna (var. *perviridis*) and turnip (var. *rapa*) lines, respectively.

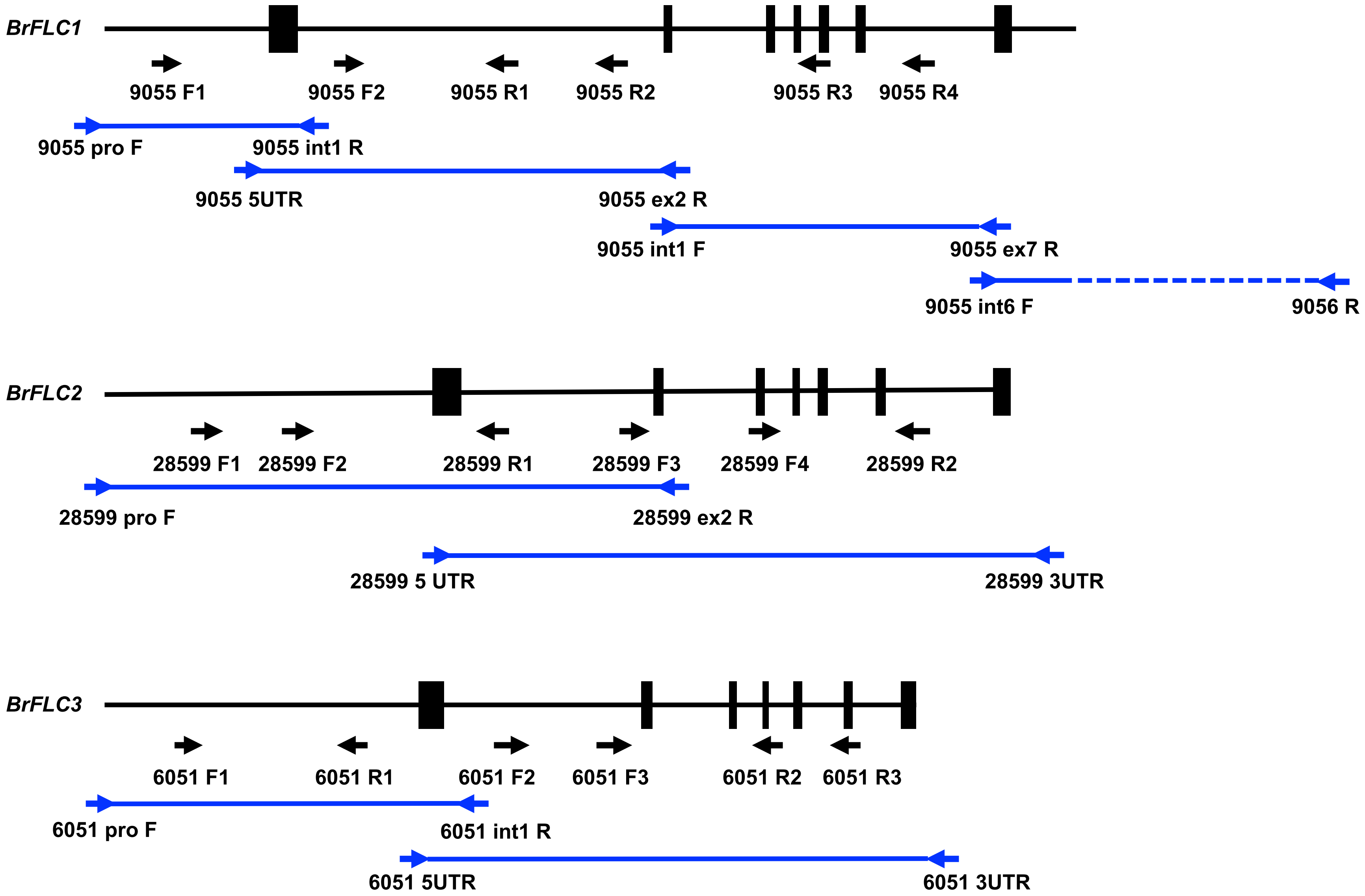

Supplementary Figure 2. Position of primers used for determining the *BrFLC1*, *BrFLC2*, and *BrFLC3* in BRA2209. Blue lines represent the regions amplified by PCR. Black arrows represent the primers used for sequencing.

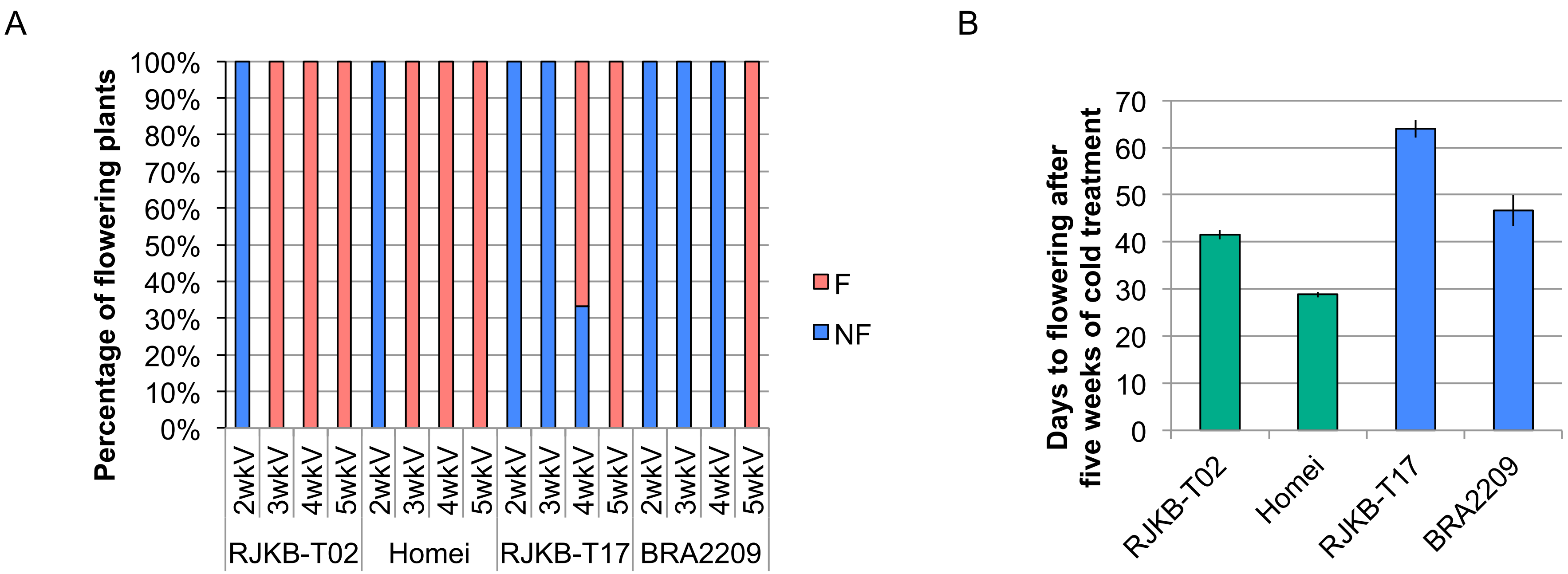

Supplementary Figure 3. The percentage of the plants that flowered after 2, 3, 4, or 5 weeks of cold treatment (A). 'F' represents the percentage of plants that flowered within 100 days after cold treatment. 'NF' represents the percentage of plants that did not flower after more than 100 days after cold treatment. (B) The days to flowering after five weeks of cold treatment. More than four plants were examined.

```
                    10         20         30         40         50         60         70         80         90        100
BrFRIa-GA  M--AVRNGSL LPAPSTREEE QPSSAMIQRR EAQATVETVP TNIETTIEQS NDPQFLKSIV DLTALAAAVD AFKRRYDELQ SHMDYIGNAI DSNLKTNGII
BrFRIb-GA  M--AFRNGSL IPP------H DPSSPTIQRG -------TVP TNTEITIEQS NHPQFLKSID DLTAFSAAVD AFKRHYDDLQ SHMDYIKNAI DSSLKSKGIT
AtFRI      MSNYPPTVAA QPTTTANPLL QRHQSEQRRR ELPKIVETES TSMDITIGQS KQPQFLKSID ELAAFSVAVE TFKRQFDDLQ KHIESIENAI DSKLESNGVV
           *     . :   *.        :  ..  :*         * . *. : ** ** :.*******  :*:*::.**: :***::*:** .*:: * *** **.*:::*:

                   110        120        130        140        150        160        170        180        190        200
BrFRIa-GA  ETAAAS---- PPPQN----- --KTATAIAC QSPPKEKSE- ----AERFCE SMWSKELRRY MFVNISERAK LIEEIPGALK LAKDPAKFVL DCIGKFYLQG
BrFRIb-GA  AESPSSRSQS PRNDA----- --SGETVAAT QSPPKETCET VAEKVERLCE LMCSKGLRRY MYSNISDRAK LIEELPAALK LAKEPANFVL ECIGKFYLQG
AtFRI      LAARNNNFHQ PMLSPPRNNV SVETTVTVSQ PSQEIVPETS NKPEGGRMCE LMCSKGLRKY IYANISDQAK LMEEIPSALK LAKEPAKFVL DCIGKFYLQG
             :  .     *  .         .  .. :  *                *:**  * ** **:* :: ***::** *:**:*.*** ***:**:*** :*********

                   210        220        230        240        250        260        270        280        290        300
BrFRIa-GA  RKAFAKDLPA ITARKVSLLI LECYLLTFDP EGEKKKKLLV SSVKDEAEAA AVAWKKRLVG EGWLGAAEAM DARGLLLLVA CFGIPESFKS MDLLDLIRQS
BrFRIb-GA  RKAYASDSHM IPARQVSLLI LESYLLMLDP ----KKPFDR VSIKDQAEAA AVAWKKRMMS EGRLAAAEAM DARGLLLLIA CFGIPSSFSS MDLFDLVRKS
AtFRI      RRAFTKESPM SSARQVSLLI LESFLLMPDR G--KGKVKIE SWIKDEAETA AVAWRKRLMT EGGLAAAEKM DARGLLLLVA CFGVPSNFRS TDLLDLIRMS
           *:*::.:     .**:***** **.:**  *       *      :**:**:* ****:**::  ** *.*** * ********:* ***:*..* *  **:**:* *

                   310        320        330        340        350        360        370        380        390        400
BrFRIa-GA  GTDEIVGALK RSPFLVPMMS GIVDSSIKRG MHIEALELVY TFGMEDRFSP SSILTSFLRM RKDSFERAKR QAQAPMASKT ANEKQLDALS SVMKCLEAHK
BrFRIb-GA  GAAEIAAALK RSPFLVPMMS GIVDSSIKRG KHIEALGMIY TFGIEDRFSA SSLLTSFLRM SKESFERAKQ KAQAPIAFKE ANQKFLAALL SVMKCLEAHN
AtFRI      GSNEIAGALK RSQFLVPMVS GIVESSIKRG MHIEALEMVY TFGMEDKFSA ALVLTSFLKM SKESFERAKR KAQSPLAFKE AATKQLAVLS SVMQCMETHK
           *: **..*** ** *****:* ***:******  ***** ::* ***:**:**. : :*****:*  *:******: :**:*:* * *  * * .*  ***:*:*:*:

                   410        420        430        440        450        460        470        480        490        500
BrFRIa-GA  LDPAKEVPGW QIKEQMAKLE KDIVQLDKQM EE-ARSISRM EEARSISRME EARSISIREE AAISERLYNQ QMKRPRLSEM EMPPTAAASY SPMYRDHRSF
BrFRIb-GA  LDPEREVQGW QIKEQMIKLE KDIIQLDKQM EGEARSISLM EE-------- ---------- VALTKRFYNQ QMKRPRLSDM EMPPAASSSY SSTYPDR-SF
AtFRI      LDPAKELPGW QIKEQIVSLE KDTLQLDKEM EEKARSLSLM EE-------- ---------- AALAKRMYNQ QIKRPRLSPM EMPPVTSSSY SPIYRDR-SF
           *** :*: ** *****: .** ** :****:* *  ***:*:* **                    .*:::*:*** *:****** * ****.:::** *. * *: **

                   510        520        530        540        550        560        570        580        590        600
BrFRIa-GA  PSHREGDADE ISALVSSYLG PSSGFPHRSG LMRSPEYMVP PGGLGRSVYA YDHLPPNSYS ---------- ----PVHGQR RPQEYPPPVH GQHQMPY---
BrFRIb-GA  PSHRD---NE ISALVSSYLG PSSGFPHRSS LRRSPEYLAP SSGLGRSVPA YEHLPPNSYL P-------LP GRHSPVQGQR LPGEYTPPIH GQQQIPYGLQ
AtFRI      PSQRDDDQDE ISALVSSYLG PSTSFPHRSR RSPEYMVPLP HGGLGRSVYA YEHLAPNSYS PGHGHRLHRQ YSPSLVHGQR HPLQYSPPIH GQQQLPYGIQ
           **:*:   :* ********** **:.*****     .     *  .****** * *:**.****                   *:***  * :*.**:* **:*:**

                   610        620        630
BrFRIa-GA  RLYRHSPSVE RHLALSNHRT PRNLSQDRIG GM
BrFRIb-GA  RVYRHSPSVE RYLALPKIRS PRNS------ --
AtFRI      RVYRHSPSEE RYLGLSNQRS PRSNSSLDPK --
           *:****** * *:*.*.: *: **.
```

Supplementary Figure 4. Comparison of the predicted amino acid sequences between BrFRIa (Group A), BrFRIb (Group A), and functional AtFRI (AF228499.1). The coiled-coil domains are represented in red.

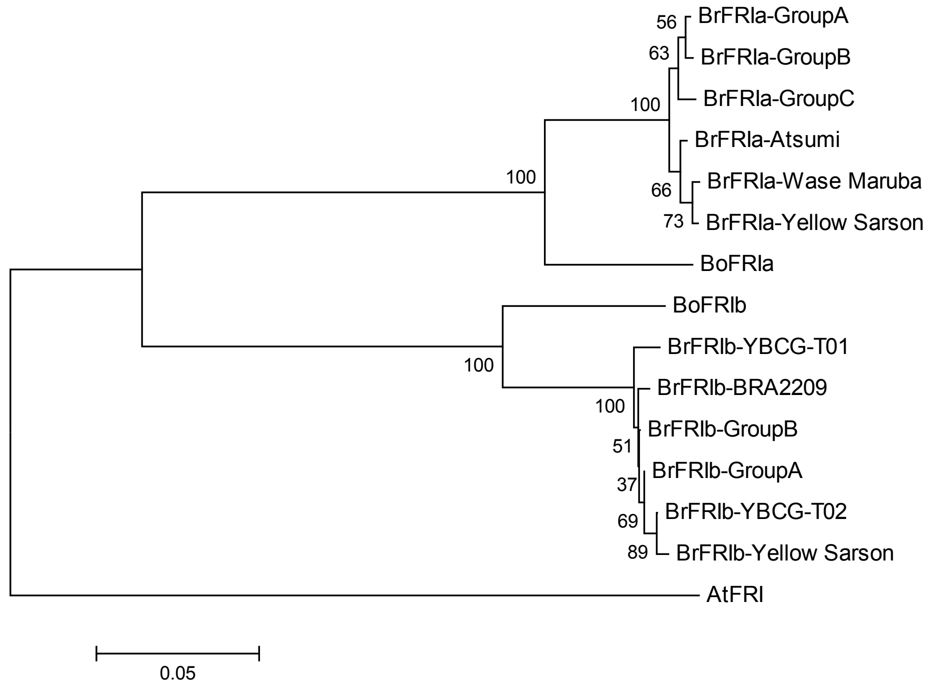

Supplementary Figure 5. Phylogenetic tree of amino acid sequences of coding region of *FRIb* genes. Bootstrap values with 1,000 replicates are indicated at the node of the phylogenetic trees. The lines involved in BrFRIa-GroupA, B, and C and BrFRIb-Group A and B are shown in Supplementary Table S4.

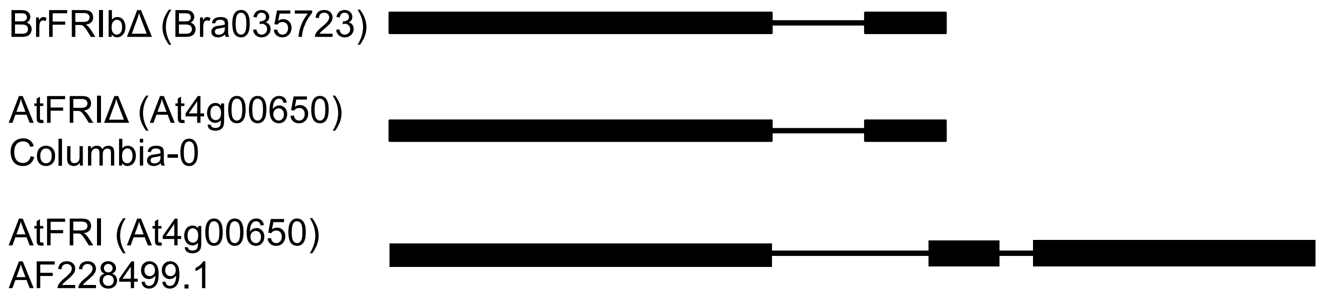


```
                10         20         30         40         50         60         70         80         90        100
BrFRIbΔ  M-AFRNGSLI PPHDPSSPTI QRG------- -------TVP TNTEITIEQS NHPQFLKSID DLTAFSAAVD AFKRHYDDLQ SHMDYIKNAI DSSLKSKGIT
AtFRI    MSNYPPTVAA QPTTTANPLL QRHQSEQRRR ELPKIVETES TSMDITIGQS KQPQFLKSID ELAAFSVAVE TFKRQFDDLQ KHIESIENAI DSKLESNGVV
         *  :        *  .:.* : **                * . *. :*** ** ::******** :*:***.**: :***::**** .*:: *:*** **.*:*:*:.

               110        120        130        140        150        160        170        180        190        200
BrFRIbΔ  AESPSSRSQS -----PRNDA SGETVAATQS PPKETCETVA EKVE--RLCE LMCSKGLRRY MYSNISDRAK LIEELPAALK LAKEPANFVL ECIGKFYLQG
AtFRI    LAARNNNFHQ PMLSPPRNNV SVETTVTVSQ PSQEIVPETS NKPEGGRMCE LMCSKGLRKY IYANISDQAK LMEEIPSALK LAKEPAKFVL DCIGKFYLQG
           : ... :.      ***:. * **..:... *.:*    .: :* *  *:** ********:* :*:****:** *:**:*:*** ******:*** :*********

               210        220        230        240        250        260        270        280        290        300
BrFRIbΔ  RKAYASDSHM IPARQVSLLI LESYLLMLDP KKPFDRVS-- IKDQAEAAAV AWKKRMMSEG RLAAAEAMDA RGLLLLIACF GIPSSFSSMD LFDLVRKSGA
AtFRI    RRAFTKESPM SSARQVSLLI LESFLLMPDR GKGKVKIESW IKDEAETAAV AWRKRLMTEG GLAAAEKMDA RGLLLLVACF GVPSNFRSTD LLDLIRMSGS
         *:*::.:* *  .******** ***:*** *   *   ::.  ***:**:*** **:**:*:**  ***** *** ******:*** *:**.* * * *:**:* **:

               310        320        330        340        350        360        370        380        390        400
BrFRIbΔ  AEIAAALKRS PFLVPMMSGI VDSSIKRGKH IEALGMIYTF GIEDRFSASS LLTSFLRMSK ESFERAKQKA QAPIAFACPF FTH------- ----------
AtFRI    NEIAGALKRS QFLVPMVSGI VESSIKRGMH IEALEMVYTF GMEDKFSAAL VLTSFLKMSK ESFERAKRKA QSPLAFKEAA TKQLAVLSSV MQCMETHKLD
         ***.*****  *****:*** *:****** * **** *:*** *:**:***:  :*****:*** *******:** *:*:**    .   .:

               410        420        430        440        450        460        470        480        490        500
BrFRIbΔ  ---------- ---------- ---------- ---------- ---------- ---------- ---------- ---------- ---------- ----------
AtFRI    PAKELPGWQI KEQIVSLEKD TLQLDKEMEE KARSLSLMEE AALAKRMYNQ QIKRPRLSPM EMPPVTSSSY SPIYRDRSFP SQRDDDQDEI SALVSSYLGP

               510        520        530        540        550        560        570        580        590        600
BrFRIbΔ  ---------- ---------- ---------- ---------- ---------- ---------- ---------- ---------- ---------- ----------
AtFRI    STSFPHRSRR SPEYMVPLPH GGLGRSVVYAY EHLAPNSYSP GHGHRLHRQY SPSLVHGQRH PLQYSPPIHG QQQLPYGIQR VYRHSPSEER YLGLSNQRSP

BrFRIbΔ  ---------
AtFRI    RSNSSLDPK
```

Supplementary Figure 6. Comparison of the predicted amino acid sequences between BrFRIbΔ, AtFRIΔ, and functional AtFRI (AF228499.1). The coiled-coil domains are represented in red.

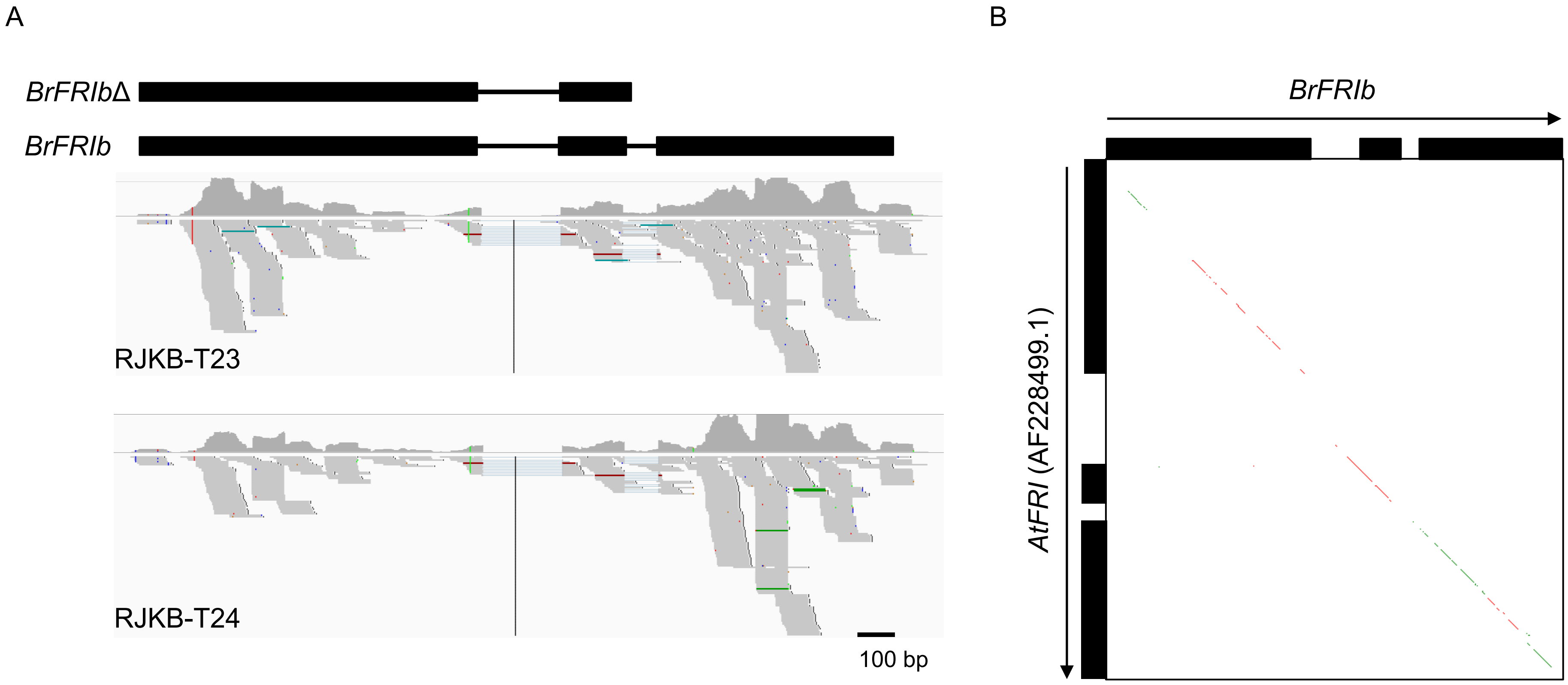

Supplementary Figure 7. The detection of new ORF in *BrFRIb*. (A) Mapping by RNA-seq reads of RJKB-T23 and RJKB-T24 against reference *B. rapa* genome.[34] (B) Harr plot analysis of the regions covering *FRI* sequences between *A. thaliana* (AF228499.1) and *B. rapa* (*BrFRIb*, reference genome). Each dot-plot shows the positions where 16 out of 20 nucleotides match in the two sequences.

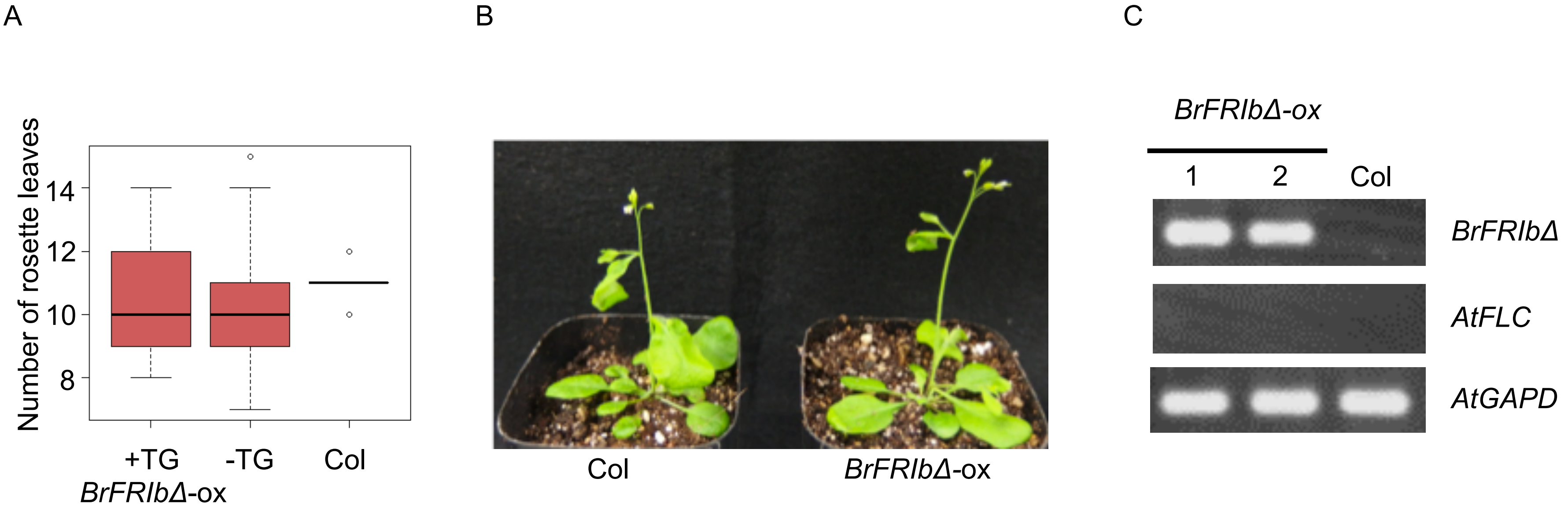

Supplementary Figure 8. Overexpression of *BrFRIbΔ* did not change the flowering time. (A) Number of rosette leaves at flowering is shown in the y-axis and the plant lines are shown in the x-axis. T_2 plants derived from three independent T_1 plants were used. There was no significant difference in rosette leaf number between T_2 plants overexpressing *BrFRIbΔ* and Col (Student's *t*-test, $p > 0.05$). -TG shows the absence of transgenes (TG). (B) Flowering-time phenotype of Col and T_2 plants overexpressing *BrFRIbΔ* (C) RT-PCR analysis of *BrFRIbΔ* and *AtFLC* transcripts using leaves without cold treatment. *AtGAPD* was used as a control to demonstrate equal RNA loading.

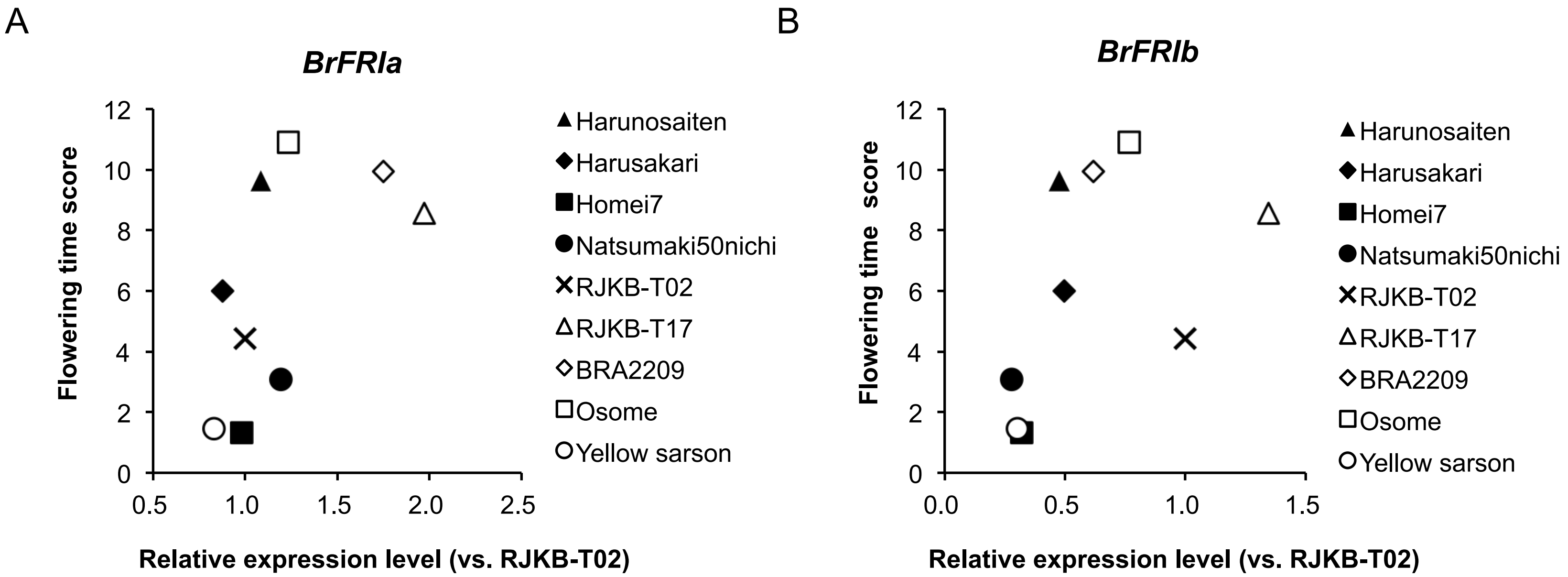

Supplementary Figure 9. The steady state expression levels of *BrFRI* is not associated with days to flower after four weeks of cold treatment. Expression level of each gene relative to *BrACTIN* is calculated, and the ratio against RJKB-T02 is used. The correlation coefficient between *BrFRIa* (A) or *BrFRIb* (B) and flowering time score are 0.59 ($p > 0.05$) or 0.49 ($p > 0.05$), respectively.

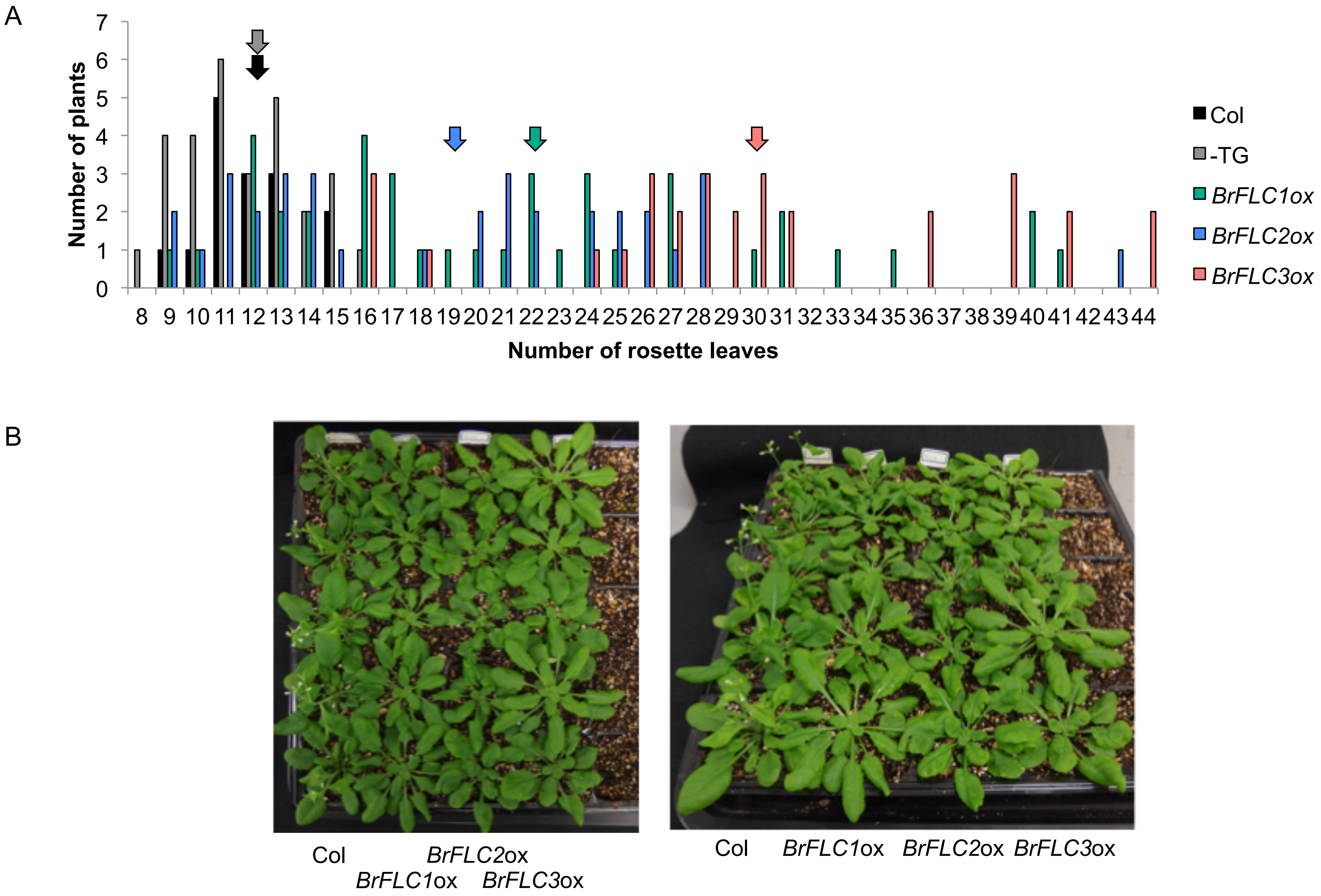

Supplementary Figure 10. Overexpressed *BrFLC1*, *BrFLC2*, or *BrFLC3* causes late flowering. (A) Distribution of the flowering time. Arrows represent the average number of rosette leaves in each line. (B) Flowering-time phenotype of Col and T_2 plants overexpressing *BrFLC1*, *BrFLC2,* or *BrFLC3*.

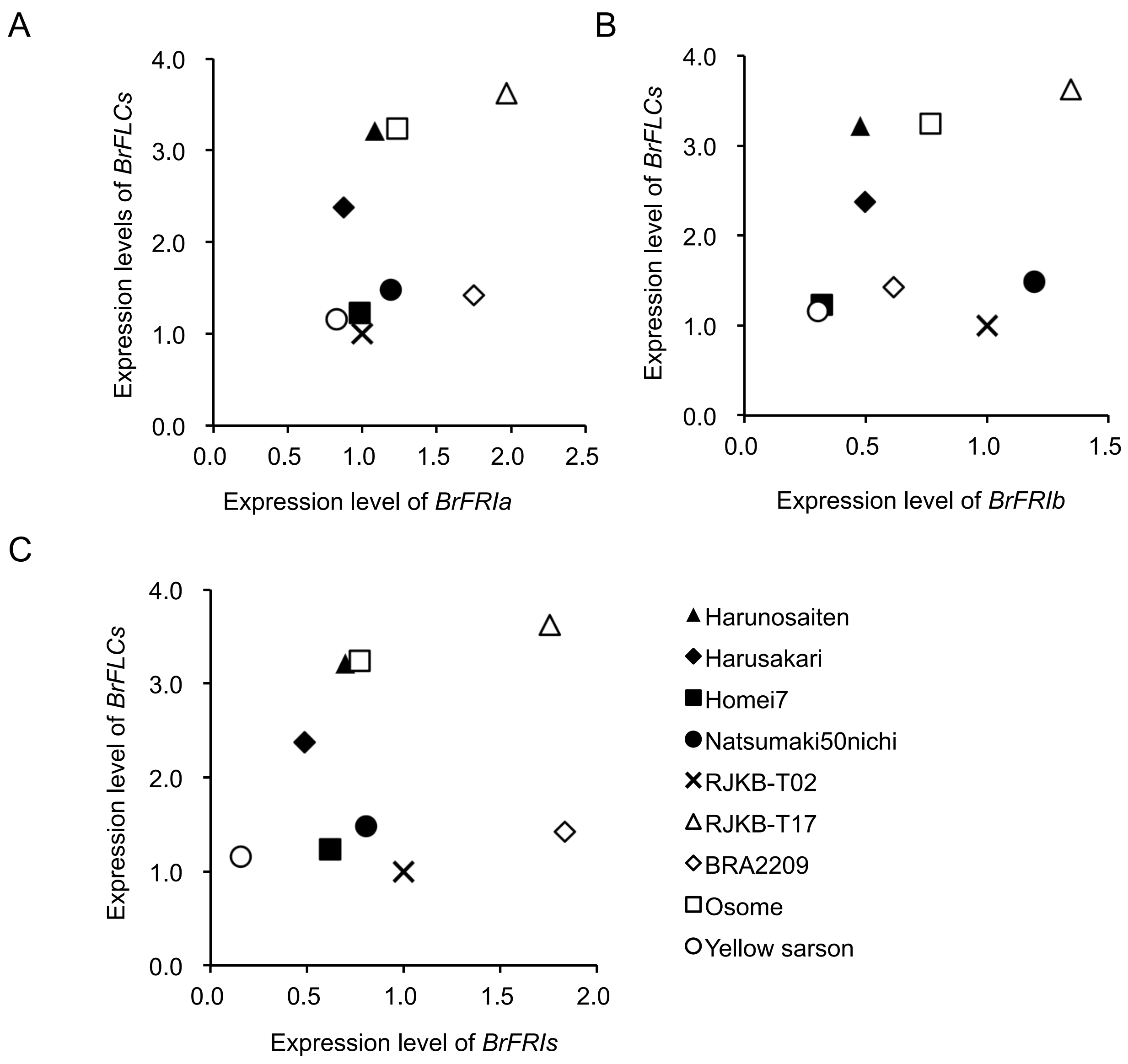

Supplementary Figure 11. The relationship between the expression levels of *BrFRIa* (A), *BrFRIb* (B) or *BrFRI*s (C) and *BrFLC*s in nine *B. rapa* lines. Expression level of each gene relative to *BrACTIN* is calculated, and the ratio against RJKB-T02 is used. The correlation coefficient between *BrFRIa*, *BrFRIb*, or *BrFRI*s and *BrFLC*s are 0.42 (p > 0.05), 0.48 (p > 0.05), or 0.23 (p > 0.05), respectively.

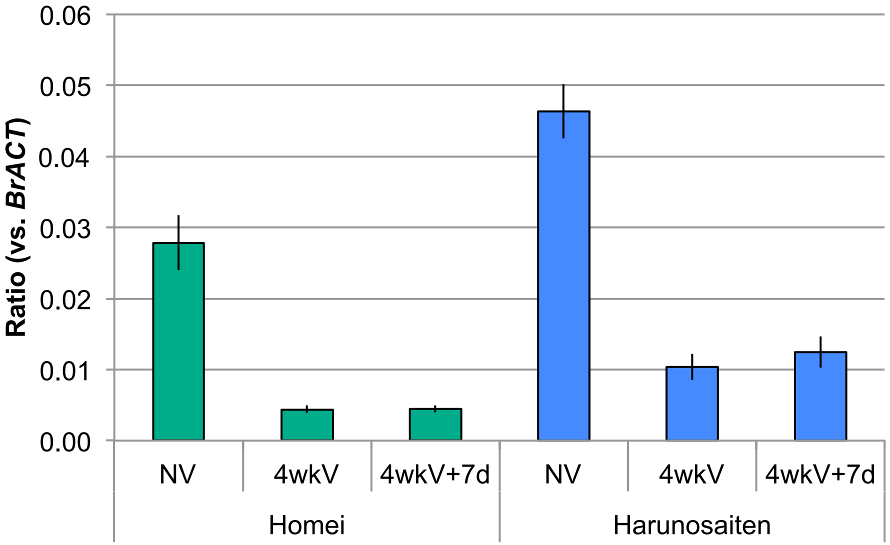

Supplementary Figure 12. Expression pattern of *BrFLC*s (*BrFLC1* + *BrFLC2* + *BrFLC3* + *BrFLC5*) in Homei and ‘Harunosaiten’ before and after four weeks of cold treatments. Expression level relative to *BrACTIN* (*BrACT*) is calculated. Data presented are the average and standard error (s.e.) from three biological and experimental replications. NV, non-vernalized; 4wkV, four weeks of cold treatment; 4wkV+7d, four weeks of cold treatment and then seven days normal growth condition.

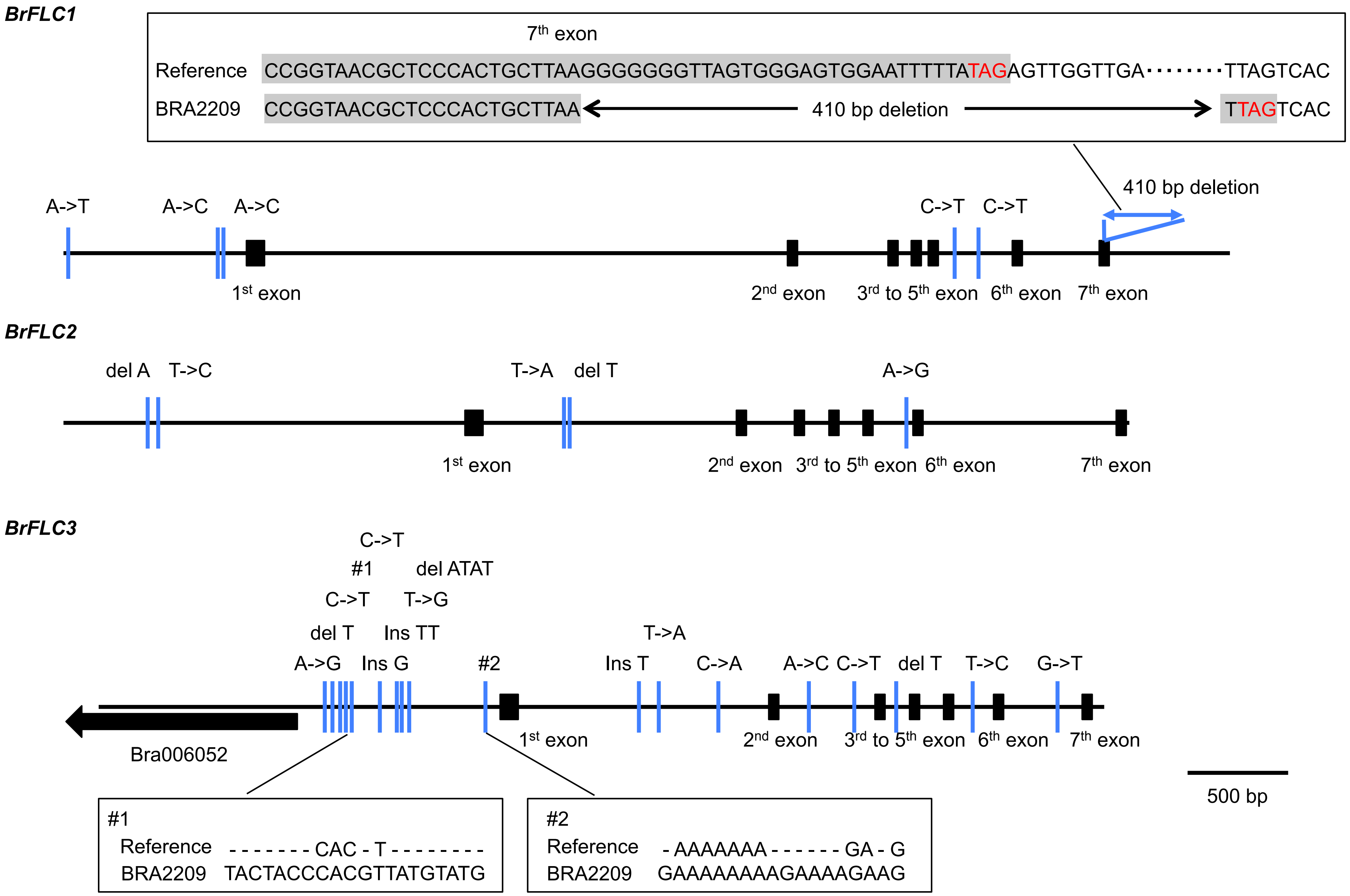

Supplementary Figure 13. Genome structure of *BrFLC1*, *BrFLC2*, and *BrFLC3* in BRA2209. Blue lines represent the position of substitutions or indels. del, deletion; ins, insertion

Supplemantary Table 1. Plant materials

Name	Type	Variety	Flowering time	*BrFRI* sequence	Genetic distance	*BrFLC* sequence	*BrFRI* expression	*BrFLC* expression	*BrFLC* expression following vernalization	ChIP
1 Harunosaiten	Commercial cultivar (Watanabe Seed Co., Ltd)	Chinese cabbage (var. *pekinensis*)	✔	✔		✔	✔	✔	✔	✔
2 Harusakari	Commercial cultivar (Watanabe Seed Co., Ltd)	Chinese cabbage (var. *pekinensis*)	✔	✔			✔	✔		
3 Natsumaki 50nichi	Commercial cultivar (Watanabe Seed Co., Ltd)	Chinese cabbage (var. *pekinensis*)	✔	✔			✔	✔		
4 Chiifu	Commercial cultivar	Chinese cabbage (var. *pekinensis*)		✔	✔					
5 Yellwo sarson	Commercial cultivar	Oilseed (var. *oleifera*)	✔	✔			✔	✔		
6 Homei	Doubled haploid line	Chinese cabbage (var. *pekinensis*)	✔	✔	✔	✔	✔	✔	✔	✔
7 BRA2209	Doubled haploid line	Turnip (var. *rapa*)	✔	✔	✔	✔	✔	✔	✔	
8 Atsumi	Doubled haploid line	Turnip (var. *rapa*)		✔	✔					
9 Kisobeni	Doubled haploid line	Turnip (var. *rapa*)		✔	✔					
10 Osome (OSD2)	Doubled haploid line	Komatsuna (var. *perviridis*)	✔	✔	✔		✔	✔		
11 Wase-Maruba	Doubled haploid line	Komatsuna (var. *perviridis*)		✔	✔					
12 RJKB-T01	Inbred line	Chinese cabbage (var. *pekinensis*)		✔	✔					
13 RJKB-T02	Inbred line	Chinese cabbage (var. *pekinensis*)	✔	✔	✔	✔	✔	✔	✔	
14 RJKB-T03	Inbred line	Chinese cabbage (var. *pekinensis*)		✔	✔					
15 RJKB-T04	Inbred line	Chinese cabbage (var. *pekinensis*)		✔	✔					
16 RJKB-T05	Inbred line	Chinese cabbage (var. *pekinensis*)		✔	✔					
17 RJKB-T06	Inbred line	Chinese cabbage (var. *pekinensis*)		✔	✔					
18 RJKB-T07	Inbred line	Chinese cabbage (var. *pekinensis*)		✔	✔					
19 RJKB-T08	Inbred line	Chinese cabbage (var. *pekinensis*)		✔	✔					
20 RJKB-T09	Inbred line	Chinese cabbage (var. *pekinensis*)		✔	✔					
21 RJKB-T10	Inbred line	Chinese cabbage (var. *pekinensis*)		✔	✔					
22 RJKB-T11	Inbred line	Chinese cabbage (var. *pekinensis*)		✔	✔					
23 RJKB-T12	Inbred line	Chinese cabbage (var. *pekinensis*)		✔	✔					
24 RJKB-T13	Inbred line	Chinese cabbage (var. *pekinensis*)		✔	✔					
25 RJKB-T14	Inbred line	Chinese cabbage (var. *pekinensis*)		✔	✔					
26 RJKB-T15	Inbred line	Chinese cabbage (var. *pekinensis*)		✔	✔					
27 RJKB-T16	Inbred line	Chinese cabbage (var. *pekinensis*)		✔	✔					
28 RJKB-T17	Inbred line	Chinese cabbage (var. *pekinensis*)	✔	✔	✔	✔	✔	✔		
29 RJKB-T18	Inbred line	Chinese cabbage (var. *pekinensis*)		✔	✔					
30 RJKB-T19	Inbred line	Chinese cabbage (var. *pekinensis*)		✔	✔					
31 RJKB-T20	Inbred line	Chinese cabbage (var. *pekinensis*)		✔	✔					
32 RJKB-T21	Inbred line	Chinese cabbage (var. *pekinensis*)		✔	✔					
33 RJKB-T22	Inbred line	Chinese cabbage (var. *pekinensis*)		✔	✔					
34 RJKB-T23	Inbred line	Chinese cabbage (var. *pekinensis*)		✔	✔					
35 RJKB-T24	Inbred line	Chinese cabbage (var. *pekinensis*)		✔	✔	✔				
36 YBCG-01	Inbred line	Komatsuna (var. *perviridis*)		✔	✔					
37 YBCG-02	Inbred line	Komatsuna (var. *perviridis*)		✔	✔					

[#] *FLC* expression levels following different duration of cold treatments were examined

Supplemantary Table 2. Criteria for evaluating flowering time

Score	Days for flowering after four weeks of cold treatment
1	46 =< x =< 50
2	51 =< x =< 55
3	56 =< x =< 60
4	61 =< x =< 65
5	66 =< x =< 70
6	71 =< x =< 55
7	76 =< x =< 80
8	81 =< x =< 85
9	86 =< x =< 90
10	91 =< x =< 95
11	96 =< x =< 100
12	101 =< x

Supplementary Table 3. Sequences of primers used in this study

Name	Primer sequences (5'-3')	
**RT-PCR/RT-qPCR**		
AtGAPD	CTCACTTGAAGGGTGGTGCT	TGGTCATGAGTCCCTCAACA
AtFLC	GTCGCTCTTCTCGTCGTCTC	TGACATTTGATCCCACAAGC
BrActin	CGGTCCAGCTTCGTCATACTCAGCC	AAATGTGATGTGGATATCAGGAAGG
BrFRIa	AGAAGCTTTTGGTTAGTTCTGTG	TCATCAGTACCACTCTGCCTAAT
BrFRIb	AACGGTCACCTTTCCTTGTC	GCTTTCTGTTTTGCCCTCTC
BrFRIa/b	GGCGGCTGCTGTTGCGTGGAAG	CTTGATACTTGAATCAACTATAC
BrFLC1/2/3/5	GACGCARYGGTCTCATYGAGAAAGC	AWCATTARTTYTGTCTTYSTAGCTC
BrFLC1	CTTGAGGAATCAAATGTCGATAA	CCCTTAAGCAGTGGGAGCGTTAC
BrFLC2	AGTAAGCTTGTGGAATCAAATTCTG	TAATTAAGYAGYGGGAGAGTYAC
BrFLC3	GTGGAATCAAATGTCGGTGG	TAATTAAGYAGYGGGAGAGTYAC
BrFLC5	TAATGTTAAAGCTTGTTGAAAGT	TAATTAAGYAGYGGGAGAGTYAC
**Sequence of DNA fragments of *BrFRI*a or *BrFRI*b**		
BrFRIa-F1/R1	GAGTCAAAAAGATATTAATAGGGCC	GAAATATCAATTACAGATCCTAAGC
BrFRIa-F2/R2	TGAACATCTCTGAGCGAGCC	ATTTCTGACAACCTTGGACG
BrFRIa-F3/R3	GGAGCAAAGAGCTCCGAAGG	GGTGAATGTCTGTATAGACG
BrFRIb-F1/R1	TACATACCTTATCTCTCCGACATC	ACTTCATCACTGATAACAACGCAGC
BrFRIb-F2/R2	CCAAGGCAATTGCACTCTAA	CAACTCGCACAATCGCTCCA
**Sequence of the full length coding region in each *FLC* paralog**		
BrFLC1	CAAAGCACTGTTGGAGACAGAAGCC	CCAACTCTATAAAAATTCCACTCCC
BrFLC2	TCAAATTAGGGCACAAAGGCTTCTC	GTCACAAGTTTTGGACTTAAGGTGG
BrFLC3	TCAAATTAGGGCACAGAGACCACTT	TTTGATCAGCCCCGTCTAACGGTGG
**Sequence of the genic region covering each *FLC***		
BrFLC1		
9055 pro F/9055 int1 R	CTCAGAAGTATTTTAAGACC	CACCGGAGGAGAAGCTGTAG
9055 5UTR / 9055 ex2 R	CAAAGCACTGTTGGAGACAGAAGCC	CCAGGCTGGAGAGAAGAGAA
9055 int1 F / 9055 ex7 R	GTTCATTCTTTCAAGGGTTAGCTG	TCGCACAAGATTACTCTTCTCC
9055 int 6 F/ 9056 R	AGATGGAGAAGAGTAATCTTGTGCG	GCAGAGGTGCGTATGATCTAGCCAT
9055 F1 / R1	TTCGATATGTAAAGGCAAAC	GAAAATTGCAACCCCTTGTC
9055 F2 / R2	ATGGCCATGAGAAATACATG	TAATCAATTTCTAACGCTGG
9055 R3 / R4	CTTTAAGGTTCTCGACAAGC	TCGCACAAGATTACTCTTCTCC
BrFLC2		
28599 pro F/28599 ex2 R	TGATTACGCCAAGCTCGGGAATATATTTCCCCAC	GACCACCCGGGGATCTCAGAGCTTTAAGATCATCAG

28599 5 UTR / 3 UTR	TCAAATTAGGGCACAAAGGCTTCTC	GTCACAAGTTTTGGACTTAAGGTGG
28599 F1 / R1	GGCTCAGTTGCAGTTTTCACC	CATACTTATCGCCGGCGGAG
28599 F2 / R2	CTTCCTACTTCAGACCTCAC	CAGAATGATGTCCAACAACCTG
28599 F3 / F4	GGCTACTAGTTGGTTCTTAG	GGTTCACACCATGAGCTACT
BrFLC3		
6051 pro F / 6051 int1 R	TGATTACGCCAAGCTGCGTCATCCTCATCACCA	GGCTAATAAAGGAAGGCACAGA
6051 5UTR/ 6051 3UTR	TCAAATTAGGGCACAGAGACCACTT	TTTGATCAGCCCCGTCTAACGGTGG
6051 F1 / R1	CAACCATGAGGACACCAGGC	TCCTTCGATTCCACTATAGG
6051 F2 / R2	TCTTCTCTTCTGTGCCTTCC	CAGTATGAAGTCAACGTACC
6051 F3 / R3	GCAATGCACTGCACTCCGGA	ATCCAAAAACCAGAATGCCC
**For Construction**		
BrFRIb	GGATCCATGGCCTTTCGTAATGGTTC	GAGCTCCTAATGTAAAGAAGGGAC
BrFLC1	GGTCTAGAATGGGGAGGAAGAAACTTGA	GCGAGCTCCTAATAAAGCAGTCGGAGAG
BrFLC2	GGTCTAGAATGGGGAGGAAGAAACTTGA	GCGAGCTCCTATAAAAATTCCACTCCCAC
BrFLC3	GGTCTAGAATGGGGAGGAAGAAACTTGA	GCGAGCTCCTAATTAAGCAGTGGGAGAG
**ChIP-qPCR**		
BrFWA	CGGCATATGATTCGTTTGTG	CCTGGTTGTGTAGCATGTGG
BrSTM	TGGAGAGTGGTTCCAACAGCACTTC	GGAGCTACTTTGTTGGTGGTGTGAC
Bra013206	GACGAGCACAAGAGTGGTGA	TAATCGCTGTCGCTGTCACT
Bra028913	CCCTGGGAGCAACTCTGTTA	GTGGGAGCAATCCTGATGAC
BrFLC1-ex1-F/R	TGGGGAGGAAGAAACTTGAA	CACCGGAGGAGAAGCTGTAG
BrFLC2-ex1-F/R	CGACAAGTCACCTTCTCCAA	AGAGGAACGGAAGCGAAAAG
BrFLC3-ex1-F/R	TTGAGAACAAAAGTAGCCGACA	GGCTAATAAAGGAAGGCACAGA
BrFLC1-ex5-F/R	TGCTTCCAAGTTTATAGCTA	CTCCTGTTTTAAAGATCAAC
BrFLC2-ex5-F/R	ATCTTACCTTGTGTCGAGAG	AAGGTCAACATGATTATCAC
BrFLC3-ex5-F/R	TCTCGTGTTTGAAAGCCTCA	TGAAGGACAACATGTTCATC

Supplementary Table 4. The amino acid sequence identities of FRIs

		BrFRIa						BrFRIb						AtFRI	BoFRIa	BoFRIb
		GA	GB	GC	A	WM	YS	GA	GB	B2	Y1	Y2	YS			
BrFRIa	Group A (GA)		99.4	98.6	98.6	98.4	98.8	63.8	63.8	64.1	63.4	64.0	63.8	57.4	87.9	64.3
	Group B (GB)	99.4		99.1	98.1	97.9	98.3	63.9	63.9	64.2	63.5	64.1	63.9	57.5	87.9	64.2
	Group C (GC)	98.6	99.1		98.4	97.8	97.8	63.9	63.9	64.1	63.5	64.1	63.9	57.1	87.7	64.2
	Atsumi (A)	98.6	98.1	98.4		98.8	98.8	64.0	63.9	64.3	63.6	64.1	64.0	56.9	88.1	64.4
	Wase Maruba (WM)	98.4	97.9	97.8	98.8		99.6	63.5	63.5	63.9	63.1	63.7	63.5	56.8	88.1	64.0
	Yellow Sarson (YS)	98.8	98.3	97.8	98.8	99.6		63.5	63.5	63.9	63.1	63.7	63.5	56.8	87.9	64.0
BrFRIb	Group A (GA)	63.8	63.9	63.9	64.0	63.5	63.5		98.6	96.8	95.8	97.3	97.0	59.9	65.4	86.3
	Group B (GB)	63.8	63.9	63.9	63.9	63.5	63.5	98.6		98.2	97.2	98.4	98.0	59.9	65.4	87.4
	BRA2209 (B2)	64.1	64.2	64.1	64.3	63.9	63.9	96.8	98.2		97.5	98.7	98.4	59.7	65.6	86.7
	YBCG-T01 (Y1)	63.4	63.5	63.5	63.6	63.1	63.1	95.8	97.2	97.5		97.7	97.3	59.4	65.0	85.8
	YBCG-T02 (Y2)	64.0	64.1	64.1	64.1	63.7	63.7	97.3	98.4	98.7	97.7		99.6	59.7	65.6	86.3
	Yellow Sarson (YS)	63.8	63.9	63.9	64.0	63.5	63.5	97.0	98.0	98.4	97.3	99.6		59.5	65.4	86.2
AtFRI		57.4	57.5	57.1	56.9	56.8	56.8	59.9	59.9	59.7	59.4	59.7	59.5		59.0	57.8
BoFRIa		87.9	87.9	87.7	88.1	88.1	87.9	65.4	65.4	65.6	65.0	65.6	65.4	59.0		66.6
BoFRIb		64.3	64.2	64.2	64.4	64.0	64.0	86.3	87.4	86.7	85.8	86.3	86.2	57.8	66.6	

BrFRIa-Group A; Homei, Harusakari, Harunosaiten, Natsumaki 50nichi, Chiifu, RJKB-T01, T02, T03, T05, T06, T07, T08, T09, T10, T11, T12, T16, T17, T18, T19, T20, T21, T22, T23, T24

BrFRIa-Group B; Osome, YBCG-01, 02, RJKB-T04, 13, 14, 15

BrFRIa-Group C; BRA2209, Kisobeni

BrFRIb-Group A; Homei, Harusakari, Harunosaiten, Natsumaki 50nichi, Atsumi, Chiifu, Kisobeni, Osome, Wase Maruba, RJKB-T01, T03, T05, T06, T07, T08, T09, T10, T12, T13, T14, T16, T18, T19, T20, T21, T22, T23, T24

BrFRIb-Group B; RJKB-T02, T04, T11, T15, T17
